# Identification and Characterization of an Early Leaf Senescence Gene *ELS1* in Soybean

**DOI:** 10.3389/fpls.2021.784105

**Published:** 2021-12-16

**Authors:** Hiroshi Yamatani, Titnarong Heng, Tetsuya Yamada, Makoto Kusaba, Akito Kaga

**Affiliations:** ^1^Institute of Crop Science, National Agriculture and Food Research Organization, Tsukuba, Japan; ^2^Department of Agronomy, Faculty of Agriculture at Kamphaeng Saen, Kasetsart University, Kamphaeng Saen, Thailand; ^3^Graduate School of Agriculture, Hokkaido University, Sapporo, Japan; ^4^Graduate School of Integrated Sciences for Life, Hiroshima University, Higashi-Hiroshima, Japan

**Keywords:** soybean, chlorophyll, CaaX-like protease, NGS-based bulked segregant analysis, GmBCM1

## Abstract

Early leaf senescence phenotype in soybean could be helpful to shorten the maturation period and prevent green stem disorder. From a high-density mutation library, we identified two early leaf senescence soybean mutant lines, *els1-1* (*early leaf senescence 1*) and *els1-2.* The chlorophyll contents of both *els1-1* and *els1-2* were low in pre-senescent leaves. They degraded rapidly in senescent leaves, revealing that *ELS1* is involved in chlorophyll biosynthesis during leaf development and chlorophyll degradation during leaf senescence. The causal mutations in *els1* were identified by next-generation sequencing-based bulked segregant analysis. *ELS1* encodes the ortholog of the *Arabidopsis* CaaX-like protease BCM1, which is localized in chloroplasts. Soybean *ELS1* was highly expressed in green tissue, especially in mature leaves. The accumulation of photosystem I core proteins and light-harvesting proteins in *els1* was low even in pre-senescent leaves, and their degradation was accelerated during leaf senescence. These results suggest that soybean *ELS1* is involved in both chlorophyll synthesis and degradation, consistent with the findings in *Arabidopsis* BCM1. The gene *els1,* characterized by early leaf senescence and subsequent early maturation, does not affect the flowering time. Hence, the early leaf senescence trait regulated by *els1* helps shorten the harvesting period because of early maturation characteristics. The *els1-1* allele with weakly impaired function of *ELS1* has only a small effect on agricultural traits and could contribute to practical breeding.

## Introduction

Soybean (*Glycine max*) is an essential crop for oil production, human consumption, and livestock feed. As with many crops, adjusting the flowering and maturation time in soybean breeding is very important for the target adaptation area. Early soybean harvesting is one of the critical agricultural processes that might increase yields, other factors being double-cropping systems, crop rotation, and cultivation at high latitudes. However, most of the early maturation genes that have been isolated so far shorten the maturation by accelerating the flowering time (Liu et al., 2008; Watanabe et al., 2009, 2011; Xia et al., 2012; Lu et al., 2020). To diversify soybean harvest time, early maturing genetic resources with causal genes that do not affect flowering time are required, but there have been no reports so far. In addition, soybean harvest is often affected by the green stem disorder (GSD), in which chlorophyll degradation is suppressed without leaf senescence during harvest (Phillips et al., 1984; Hobbs et al., 2006). GSD reduces seed quality because mechanical harvesting contaminates seeds with leaf and stem juices (Hill et al., 2006). Since early chlorophyll degradation accelerated early leaf senescence in *Arabidopsis* (Ono et al., 2019), controlling chlorophyll degradation might help adjust soybean harvest time without affecting the flowering time and prevent GSD. However, little about the effects of changes in chlorophyll metabolism on agricultural traits has been known.

Chlorophyll is an essential molecule for capturing light during photosynthesis and is synthesized during leaf development and degraded during leaf senescence. Higher plants have two types of chlorophyll: chlorophyll *a* (Chl *a*) and chlorophyll *b* (Chl *b*). Chl *a* consists of photosystem I (PSI), photosystem II (PSII), and cytochrome *b_6_f*. Chl *b* is present only in the PSI-associated light-harvesting complex II (LHCI) and the PSII-associated light-harvesting complex II (LHCII). Chlorophyll-binding proteins such as PSI and PSII convert light energy into chemical energy during light reactions. The subunits of PSI, PSII, LHCI, and LHCII are encoded by *Psa*, *Psb*, *Lhca*, and *Lhcb*, respectively.

Free chlorophyll and its intermediate forms react with light to produce harmful reactive oxygen species, ultimately leading to cell death (Apel and Hirt, 2004). Hence, both chlorophyll synthesis and degradation are strictly regulated at the genetic level. The first step of chlorophyll synthesis begins with synthesizing protoporphyrin IX, a chlorophyll precursor, from glutamyl-tRNA^Glu^ *via* several steps. Then, protoporphyrin IX is converted to Mg-protoporphyrin IX (MgP) by magnesium chelatase (MgCh). MgCh is composed of three subunits: GENEMOS UNCOUPLED 5 (GUN5)/CHLH, CHLD, and CHLI, and is activated by GUN4 (Gibson et al., 1995; Larkin et al., 2003; Davison et al., 2005; Verdecia et al., 2005). Finally, MgP is converted to Chl *a via* several steps. Chl *b* is converted from Chl *a* by the chlorophyll *a* oxygenase (CAO; Tanaka et al., 1998).

The first step of chlorophyll degradation begins with the conversion of Chl *a* to pheophytin *a* by magnesium dechelatase STAY-GENE/NON-YELLOWING 1 (SGR/NYE1; Armstead et al., 2007; Kusaba et al., 2007; Park et al., 2007; Ren et al., 2007; Sato et al., 2007; Shimoda et al., 2016). Subsequently, pheophytin *a* undergoes several steps to open the porphyrin ring and finally becomes colorless. Chl *b* is converted to Chl *a* by the Chl *b*-degrading enzymes NON-YELLOW COLORING 1 (NYC1) and NYC1-LIKE (NOL) and gets degraded *via* the Chl *a* degradation pathway (Kusaba et al., 2007; Sato et al., 2009). In addition, PSII subunit proteins NON-YELLOW COLORING 4/THYLAKOID FORMATION 1 (NYC4/THF1) and cytG/PsbM control the degradation of chlorophyll-binding proteins (Huang et al., 2013; Yamatani et al., 2013; Kohzuma et al., 2017).

Recently, a chloroplast-localized CaaX-like protease BALANCE of CHLOROPHYLL METABOLISM (BCM), which regulates chlorophyll synthesis and degradation, was isolated from *Arabidopsis thaliana* (Wang et al., 2020). *BCM1* and its paralog *BCM2* are also present in *Arabidopsis*. The functions of BCM1 and BCM2 overlap, and their double mutants show phenotypes of pale green leaves during leaf development and early chlorophyll degradation during leaf senescence. BCM1 is localized in thylakoid membranes and interacts with GUN4 and SGR to control chlorophyll synthesis and degradation (Wang et al., 2020). BCM1 has also been reported as an Mg^2+^ transporter and controls chlorophyll synthesis (Zhang et al., 2020). BCM1 is considered a factor that regulates chlorophyll synthesis and degradation at the protein level. Soybean has two copies of the *Arabidopsis BCM* ortholog. One of them has been reported to be the classical *G* gene that determines the color of the green seed coat in soybeans (Wang et al., 2018). The *G* gene has lost its function during domestication from wild soybean (*G. soja*) to cultivated soybean. Modern soybeans with yellow seed coat are the *g* mutant, and the yellow seed coat is assumed to be caused by reduced chlorophyll synthesis and/or increased chlorophyll degradation due to the loss of function of the *G* gene. In the present study, we isolated and physiologically characterized novel early leaf senescence mutants *els1* (*early leaf senescence 1*) in soybean and confirmed that *ELS1* encodes a paralog of the *G* gene, an ortholog of *Arabidopsis BCM1*, by using next-generation sequencing (NGS)-based bulk DNA analysis.

## Materials and Methods

### Plant Materials and Cultivation Conditions

Two mutant lines *els1-1* and *els1-2* that exhibit an early chlorophyll degradation phenotype were identified in the high-density mutant library by ethyl methanesulfonate (EMS) treatment twice (Tsuda et al., 2015). The mutants *els1-1* and *els1-2* were backcrossed with the wild-type (WT) cultivar Enrei, and *els1-1*, *els1-2*, and WTs were used in the experiment. The plants were cultivated in a field or a greenhouse 2018–2021 at NARO (36°20' N, 140°110' E) in Tsukuba City, Ibaraki Prefecture, Japan. Field cultivation was carried out in row-plots with 80 cm row spacing and 15 or 30 cm inter-plant spacing. Pot cultivation was carried out using a 30 cm diameter pot of Nippi Horticultural Land No. 1 soil (Kumiai Nippi Engeibaido No. 1. Nihon Hiryo, Japan). Dark treatment of soybean was performed as described by Kohzuma et al. (2017). The soybean stage was determined according to Fehr et al. (1971).

### Measurement of Photosynthesis-Related Parameters

Leaf chlorophyll content was non-destructively measured using a SPAD-502 Plus instrument (Konica-Minolta, Japan). The photosynthetic pigment was extracted with 80% acetone after crushing the leaves with liquid nitrogen. Chl *a* and Chl *b* contents were determined using the method described by Porra et al. (1989). The carbon assimilation rate was measured in sunlight using the Rapid Photosynthesis Measuring System MIC-100 (Masa International, Japan; Tanaka et al., 2021).

### NGS-Based Bulked Segregant Analysis

Genomic DNA was extracted from fresh leaves according to the method described by Tsuda et al. (2015). DNA bulks for next-generation sequencing (NGS)-based bulk DNA analyses were prepared from the *els1-2* × WT F_3_ population. DNA from 20 WT and 20 mutant individuals from each F_3_ family of WT and mutant types were bulked. The mutant and WT DNA bulks were sequenced on an Illumina HiSeq X10 platform (Illumina Inc., San Diego, CA, USA) at Macrogen Inc. (Seoul, Republic of Korea). A 150 bp paired-end library was constructed using genomic DNA following the TruSeq™ DNA PCR Free protocol for a 350 bp insert (Illumina). NGS-based bulked segregant analysis was conducted following Dougherty et al. (2018) protocol with slight modifications. The obtained data were trimmed and mapped to the reference genome Gmax_275_v2.0 using CLC Genomics Workbench ver.11 (CLC Bio, Denmark) with the following parameters: adaptor trim, ambiguous limit two, quality limit 0.01, removal of three 5'- and 3'-terminal nucleotides, discard read pairs with a minimum number of nucleotides less than 50 bp, no global alignment, no masking mode, linear gap cost, no auto-detect paired distances, match score one, mismatch cost two, deletion cost three, insertion cost three, length fraction 0.9, and similarity fraction 0.96. Variants against the reference genome were identified in the aligned reads with basic variant detection module ver.2.0 with the following parameters: ignore non-specific matches and broken read pairs, minimum read coverage ten, minimum count three, base quality filter, neighborhood radius five, minimum central quality 20, and minimum neighborhood quality 15. A shared variant track was prepared from the variant tracks for WT and mutant bulks by using the identified shared variants module ver to collect all variations between bulks and reference. 1.2 with a frequency parameter of 1%. Then, the variant frequency in the aligned reads of each bulk was re-calculated using the identified known mutations from mappings module ver.1.1 against the shared variant track with the following parameters: minimum coverage one, detection frequency 0.05, ignore broken pairs, and ignore non-specific matches. In addition, all positions overlapping with known gene annotations and the resulting amino acid changes or splicing site changes were searched using the amino acid changes module ver. 2.5 and the splice site effect predicted by module ver.1.4. Finally, variant filtering was performed according to the variant frequency of more than 75% for mutant bulk, less than 25% for WT bulk, and a variant with amino acid change.

The mutation site of *ELS1* in *els1-*1 was determined by Sanger sequencing using a SupreDye v3.1 Cycle Sequencing Kit (EdgeBioSystems, USA) and an ABI 3500xl genetic analyzer (Thermo Fisher Scientific, United States) according to the manufacturers’ instructions. Primers used for Sanger sequencing are shown in Supplemental Table 1. To confirm co-segregation between the causative mutations of *els1-1* and *els1-2* and their phenotype in the segregating populations, derived cleaved-amplified polymorphic sequence (dCAPS) markers with amplified fragment lengths of approximately 200 bp were designed. Primer pairs and restriction enzymes for the dCAPS markers are shown in Supplemental Table 2. We investigated the genotype and phenotype co-separation of 120 individuals in the *els1-1* × Enrei F_2_ population and 40 individuals in the *els1-2* × Enrei F_3_ population.

### RNA Extraction and Quantitative RT-PCR (qRT-PCR)

Total RNA was isolated from soybean tissues using TRI Reagent (MOR, United States) or RNeasy (Qiagen, Netherlands). According to the manufacturer’s protocol, first-strand cDNA was synthesized from 500 ng total RNA using ReverTra ACE qPCR RT Master Mix with gDNA Remover (TOYOBO, Japan). The synthesized cDNA was diluted tenfold and used as a template for qRT-PCR. The qRT-PCR was performed using Kapa SYBR Fast qPCR Kit (Kapa Biosystems, USA) and the ViiA7 real-time PCR system (Thermo Fisher Scientific). PCR conditions were performed according to the protocol. The primer pairs used for qRT-PCR are listed in Supplemental Table 3.

### Protein Analysis

For bule native PAGE analysis, fresh soybean leaves were crushed with liquid nitrogen and then suspended in homogenizing buffer (50 mM HEPES-KOH, pH 7.8, 400 mM sucrose, 10 mM NaCl, and 2 mM MgCl_2_). Homogenates were filtered through a two-layer Miracloth and centrifuged at 5000 × *g* for 10 min at 4°C. The obtained thylakoid membrane pellets were suspended in 25BTH20G buffer (50 mM Bis-Tris–HCl, pH 7.0, 20% glycerol) and centrifuged at 14000 rpm for 1 min at 4°C. The thylakoid membranes corresponding to 100 mg fresh weight (FW) were dissolved in 200 μl of 1% β-dodecyl-maltoside in the dark and on ice. Solubilized thylakoid membrane proteins were electrophoresed using Native PAGE ™ 4 to 16%, Bis-Tris, 1.0 mm, Mini Protein Gel (Thermo Fisher Scientific), according to Yamatani et al. (2018). For 2D-SDS PAGE, the excised strip-shaped gel was heat-denatured in equilibration buffer (250 mM Tris, pH 6.8, 4% SDS, 1% dithiothreitol [DTT], 0.1% bromophenol blue [BPB], and 10% glycerol) at 70°C for 5 min and then shaken for 20 min at room temperature. Electrophoresis was performed using an acrylamide gel containing 6 M urea.

For western blot and SDS-PAGE analysis, total protein was extracted from 100 mg FW soybean leaves with 400 μl of 2× SDS buffer (0.125 M Tris, pH 6.8, 4% SDS, 4% mercaptoethanol, 1% BPB, and 20% glycerol) followed by heat denaturation at 100°C for 5 min and then 10-fold diluted with 1× SDS buffer (62.5 mM Tris, pH 6.8, 2% SDS, 2% mercaptoethanol, 0.5% BPB, and 10% glycerol). The extracted proteins were electrophoresed on an acrylamide gel. Protein transfer was performed using the Trans-Blot® Turbo ™ Transfer System (Bio-Rad, Hercules, CAUSA) or Mini Trans-Blot® Cell (BIO-RAD). Antibodies against D2, Lhca1-Lhca4, Lhcb1, Lhcb3, Lhcb4, PsaH, and PsaL for western blotting were purchased from Agrisera (Agrisera, Sweden). Antibodies against D1 (Kato et al., 2012), VAR2 (Sakamoto et al., 2003), and TIC110 (Kikuchi et al., 2013) were also used. Protein detection was performed using an ECL Prime Western Blotting Detection System (Cytiva, USA) and ImageQuant LAS 4000 mini (Cytiva). SDS-PAGE gels were stained with CBB Stain One Super (Ready to Use) (Nacalai, Japan).

### Accession Numbers

The following soybean genes were used: *GmACTIN* (Glyma.08G182200), *ELS1*/*GmBCM1* (Glyma.11G043400), *GmBCM2* (Glyma.01G198500), *GmSGR1* (Glyma.11G027400), *GmNYC1* (Glyma.09G191200), *GmNAC01* (Glyma.15G254000), *GmSAG15* (Glyma.06G162200), *GmLhca1* (Glyma.02G064700), *GmLhca2* (Glyma.16G016100), *GmLhcb1* (Glyma.16G165500), *GmLhcb2* (Glyma.02G305400), *GmPsaH* (Glyma.07G019700), and *GmPsaL* (Glyma.18G241700).

## Results

### Pale Green and Early Leaf Senescence Phenotype of *els1*

**Figure 1:**
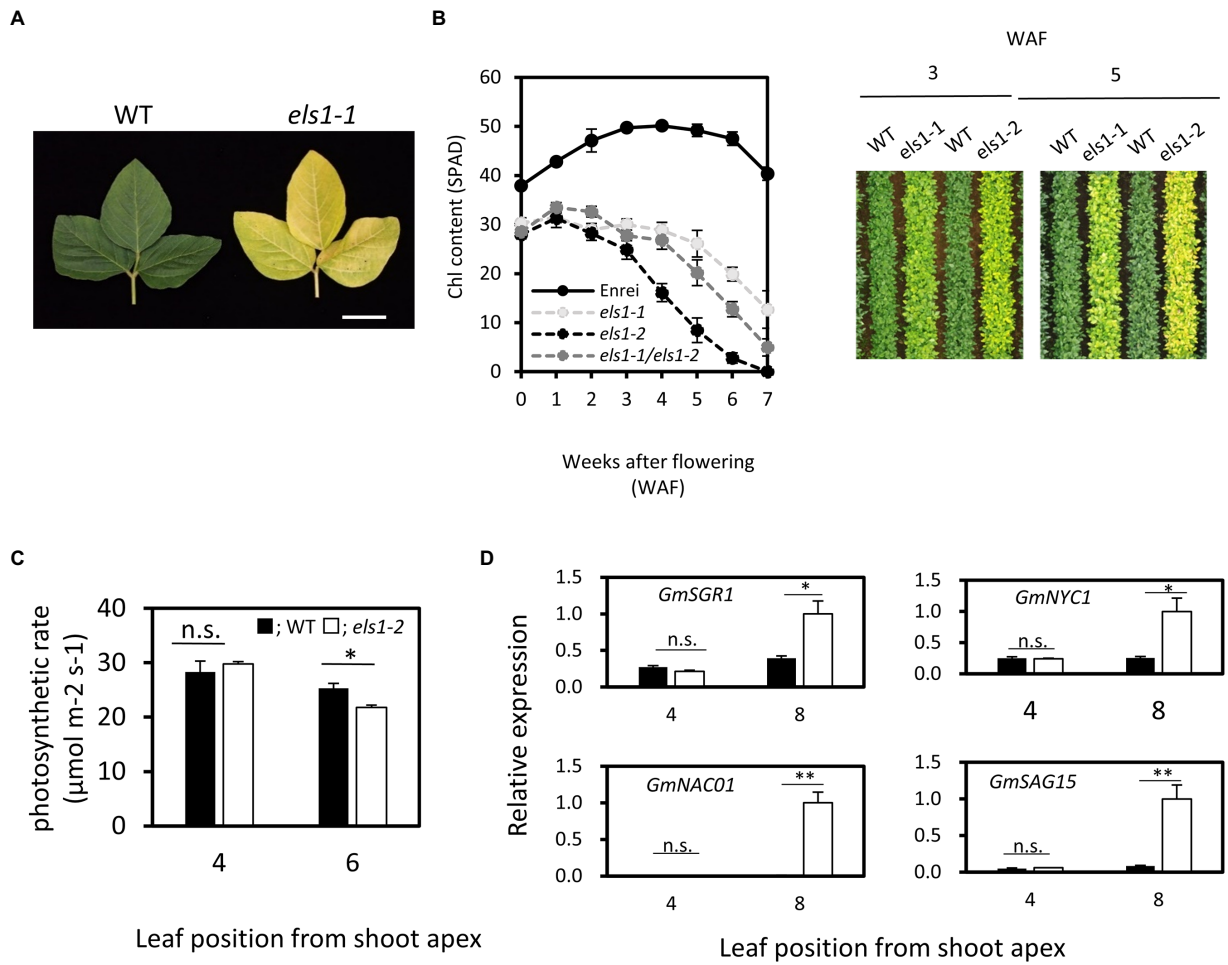
Physiological characterization of *els1* during natural leaf senescence. **(A)** *els1-1* showed an early yellowing phenotype compared to WT during natural leaf senescence. The photo shows the upper 4th leaves at 5 weeks after flowering. The scale bar indicates 5 cm. **(B)** Time course of chlorophyll content in *els1* during natural during natural leaf senescence. The chlorophyll content (SPAD) of upper 4th leaves was measured from the flowering. Solid lines; Enrei, light gray dotted lines; *els1-1*, dotted lines; *els1-2*, dark gray dotted lines; heterozygote lines between *els1-1* and *els1-2*. The error bars indicate the standard error (SE) (n = 6 biological replicates). The right panels were aerial photographs taken by a drone at 3 and 5 weeks after flowering. **(C)** The carbon assimilation rates of pre-senescent (the upper 4th leaves) and senescent (the upper 6th leaves) leaves (n = 5 biological replicates) of WT and *els1-2*. Black bars indicate WT, white bars indicate *els1-2*. Error bars indicate standard error (SE). *p < 0.05, **p < 0.01, n.s., not significant (Student’s t-test). **(D)** Expression pattern of senescence-inducible genes in WT and *els1-2* during natural leaf senescence. Total RNA from pre-senescent (the upper 4th leaves) and senescent (the upper 8th leaf) leaves of WT and *els1-2* were examined. Expression levels were standardized using *GmACTIN* (n = 6 biological replicates).

The two early leaf senescence mutants, *els1-1* and *els1-2*, were isolated from a mutant library in which high-density mutations were induced by EMS treatments (Tsuda et al., 2015). Unlike the WT cultivar., Enrei, the upper 4th leaves of *els1-1* at 5 weeks after flowering (5 WAF) exhibited an early yellowing phenotype (Figure 1A). The chlorophyll content of the upper 4th leaves of *els1-1* and *els1-2* had lower chlorophyll content than the WT cultivar Enrei at 0 WAF (SPAD: Enrei, 37.92 ± 0.69; *els1-1*, 30.30 ± 1.05; *els1-2*, 28.50 ± 0.91) (Figure 1B). Chlorophyll in both *els1* mutants was degraded earlier than Enrei at 6 WAF (SPAD value: Enrei, 47.52 ± 1.35; *els1-1*, 19.88 ± 1.41; *els1-2*, 12.75 ± 1.05). Since the chlorophyll content of *els1-2* diminished faster than *els1-1,* the *els1-2* phenotype was more severe than the *els1-1* phenotype. Aerial photographs acquired by a drone at 3 and 5 WAF also showed that the leaves of *els1* exhibited an early chlorophyll degradation phenotype (Figure 1B). These results indicate that *els1* shows phenotypes of pale green in pre-senescent leaves and early chlorophyll degradation in senescent leaves.

To determine if the causative genes of *els1-1* and *els1-2* are at the same locus, a complementation test was performed by crossing these plants. Heterozygote plants between *els1-1* and *els1-2* showed the identical phenotypes of pale green and early chlorophyll degradation as both parents, suggesting that mutations of *els1-1* and *els1-2* occurred in the same gene (Figure 1B).

The content of both Chl *a* and Chl *b* in *els1-2* was lower than that in the WT at 0 WAF (Chl *a*: WT, 2.35 ± 0.18 nmol mg^−1^ FW; *els1-2*, 1.57 ± 0.07 nmol mg^−1^ FW; Chl *b*: WT, 0.58 ± 0.04 nmol mg^−1^ FW; *els1-2*, 0.32 ± 0.02 nmol mg^−1^ FW; Supplemental Figure 1). The Chl *a*/*b* ratio of *els1-2* was higher than that of the WT at 0WAF (WT, 4.06 ± 0.02; *els1-2*, 4.79 ± 0.06, *p* < 0.01), indicating that the accumulation of Chl *b* was more significantly reduced in *els1-2*. In addition, since the decreasing rate of chlorophyll content of *els1-2* at 4 WAF was higher than that of the WT, early chlorophyll degradation occurred in *els1-2* (Chl *a*: WT, 3.39 ± 0.11 nmol mg^−1^ FW; *els1-2*, 0.88 ± 0.09 nmol mg^−1^ FW; Chl *b*: WT, 0.96 ± 0.03 nmol mg^−1^ FW; *els1-2*, 0.14 ± 0.01 nmol mg^−1^ FW).

Next, we examined the physiological functions of the leaves in *els1-2* during natural leaf senescence. Carbon assimilation rate of pre-senescent leaves (the upper 4th leaves) did not differ between the WT and *els1-2* (WT, 28.31 ± 1.99 μmol m^−2^ s^−1^; *els1-2*, 29.79 ± 0.41 μmol m^−2^ s^−1^). However, the carbon assimilation rate in the senescent leaves of *els1-2* (the upper 6th leaves) decreased faster than in the WT (WT, 25.34 ± 0.87 μmol m^−2^ s^−1^; *els1-2*, 21.78 ± 1.13 μmol m^−2^ s^−1^; Figure 1C). Additionally, the expression levels of senescence-inducible genes (*GmSGR1*, magnesium dechelatase gene; *GmNYC1*, Chl *b* -degrading enzyme gene; *GmNAC01*, senescence-inducible transcription factor gene; *GmSAG15*, senescence-inducible marker gene) in pre-senescent leaves (the upper 4th leaves) of *els1-2* during natural leaf senescence was not significantly different from that of the WT, but that of *els1-2* in senescent leaves (the upper 8th leaves) were significantly increased compared to the WT (Figure 1D). These results also physiologically confirmed that *els1* exhibits an early leaf senescence phenotype during natural leaf senescence.

Moreover, we investigated dark-induced leaf senescence using primary leaves in *els1-2*. The results showed that *els1-2* exhibited an early chlorophyll degradation phenotype 4 days after dark incubation (4 DAD; Supplemental Figure 2A). In addition, the chlorophyll content of *els1-2* was lower than that of the WT in pre-senescent leaves (0 DAD) and decreased faster than that of the WT in senescent leaves (4 DAD; Supplemental Figure 2B). During dark treatment, the expression level of senescence-inducible genes in *els1-2* at 0 DAD was not significantly different from that of the WT, but that of *els1-2* at 4 DAD was considerably higher than that of the WT (Supplemental Figure 2C). Taken together, these results indicate that *els1* shows the phenotype of pale green leaves and early leaf senescence not only during natural leaf senescence but also during dark-induced leaf senescence.

### Isolation of the *ELS1* Gene by NGS-Based Bulked Segregant Analysis

**Figure 2:**
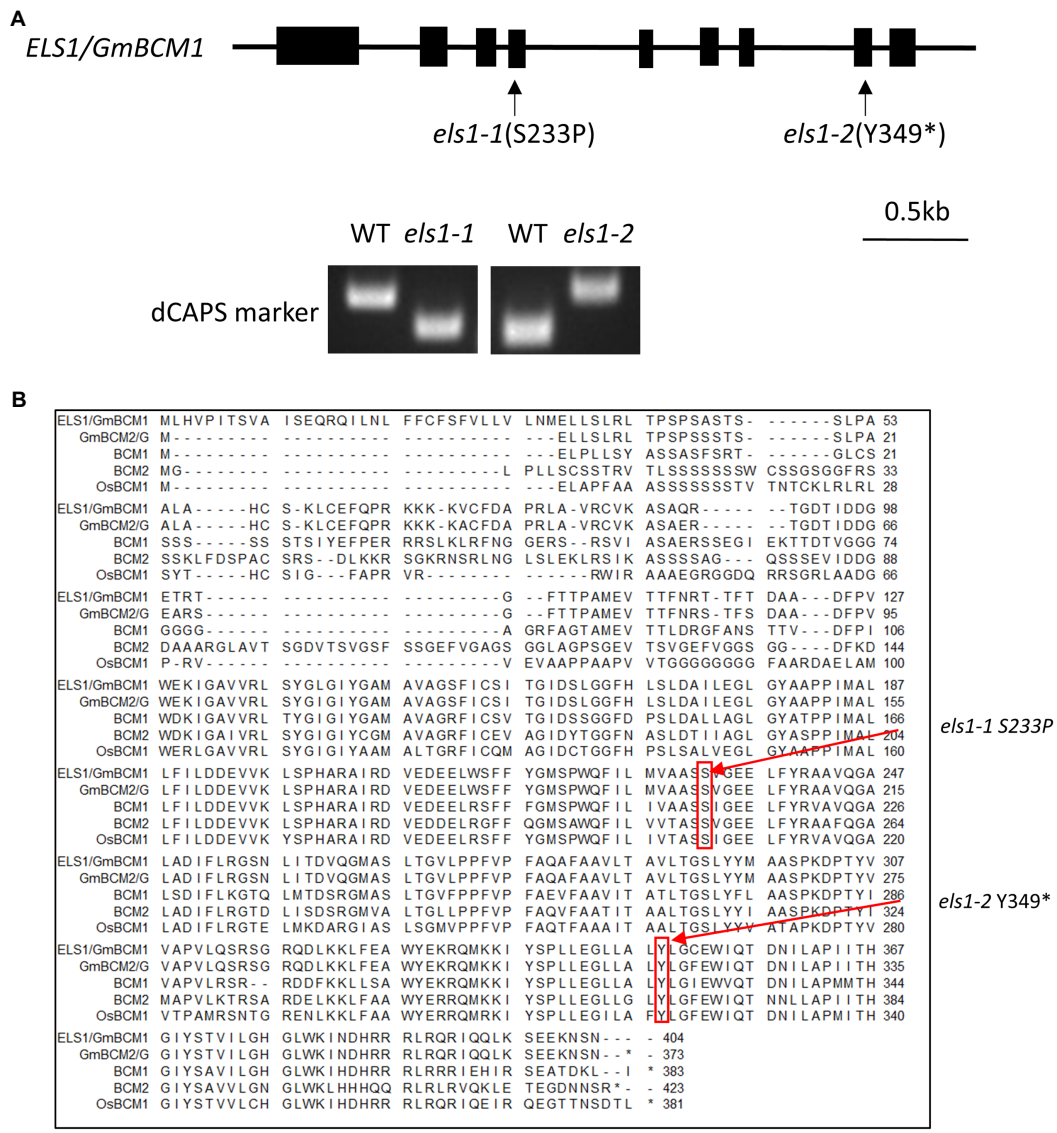
Isolation of *ELS1/GmBCM1.* **(A)** Gene structure of *ELS1*/*GmBCM1.* Black boxes indicate exons. Arrows indicate the positions of mutations. *els1-1* had an amino acid substitution from serine to proline at position 233, and *els1-2* had a nonsense mutation at position 349. The dCAPS marker was developed at the position of the base substitution. **(B)** Alignment diagram of *ELS1*/*GmBCM1.* The red arrows indicate the positions of mutations. *els1-1* and *els1-2* have single-base-substitution causing amino acid substitution in ELS1/GmBCM1. BCM1; AT2G35260 (*A. thaliana*), BCM2; AT4G17840 (*A. thaliana*), OsBCM1; Os03g0100030 (*O. sativa*). The asterisk “*” indicates a single nucleotide substitution resulting in a premature termination at the amino acid position.

The causative gene of the *els1-2* mutant was determined by NGS-based bulked segregant analysis using the F_3_ population for *els1-2*. Twenty individual plants from each F_3_ family of WT and mutant types were bulked. As a result of variant filtering of single nucleotide polymorphisms (SNPs) and indels obtained from NGS-based bulk segregant analysis in *els1-2* under three conditions (variant frequency of more than 75% in mutant bulk, variant frequency of less than 25% in WT bulk, and a variant with amino acid change), seven genes were identified as candidates. Among them, Glyma.11G043400 was considered the candidate gene for *ELS1* because the variant frequency was 100% in the mutant bulk (Supplemental Table 4). Glyma.11G043400 of *els1-2* had a base substitution from cytosine to adenine at position 1,027 bp from the start codon in the 8th exon, resulting in the formation of a premature stop codon at the 349th tyrosine (Figure 2A). As the complementation test above suggested that *els1-1* and *els1-2* are mutants at the same locus, we examined the coding region sequence of Glyma.11G043400 in *els1-1* by Sanger sequencing. We found a base substitution from thymine to cytosine in the 4th exon which resulted in an amino acid substitution from serine to proline at position 233aa (Figure 2A). Since the serine 233aa is conserved in eudicots (*A. thaliana*) to monocots (*O. sativa*), this amino acid substitution would be expected to affect protein function (Figure 2B). We analyzed genotypes of 120 individuals in the F_2_ population of *els1-1* × Enrei and 40 individuals in the F_3_ population of *els1-2* × Enrei to examine whether *els1-1* and *els1-2* mutations in Glyma.11G043400 were consistent with the early leaf senescence phenotype. The mutant alleles of *els1-1* and *els1-2* were completely co-segregated with the early leaf senescence phenotype (Supplemental Figures 3, 4), indicating that *ELS1* encodes Glyma.11G043400, the *Arabidopsis* ortholog *GmBCM1* of the chloroplast-localized CaaX-like protease, BCM1. *els1-2* is considered a null allele because it forms a premature stop codon, whereas *els1-1* is a weak allele because it has an amino acid substitution and a milder phenotype of early leaf senescence than *els1-2*.

### Expression Pattern and Light Response of *ELS1*

**Figure 3:**
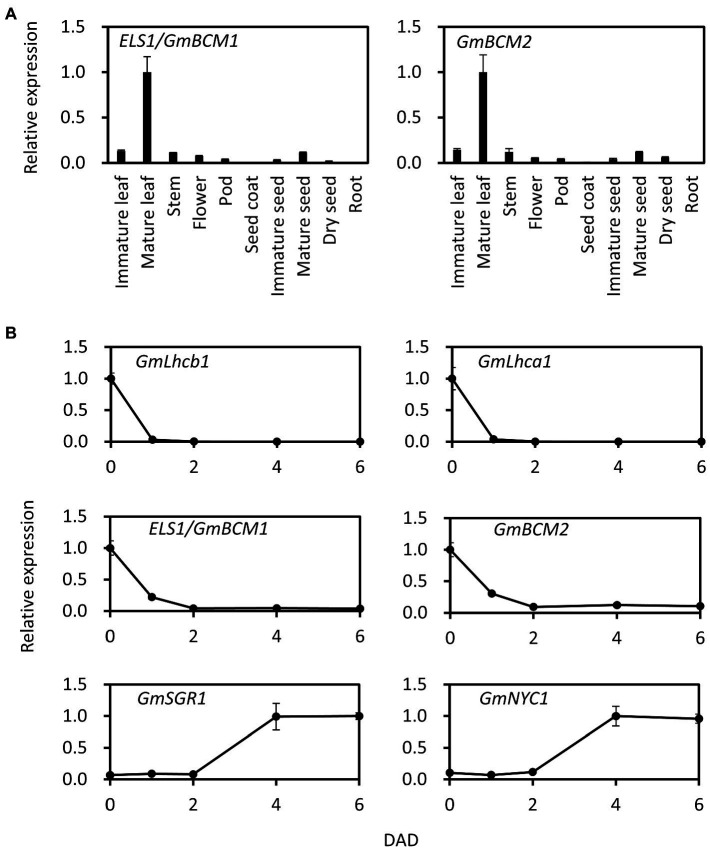
Expression pattern of *ELS1/GmBCM1* and *GmBCM2* in tissue and during dark treatment. **(A)** Total RNA from each tissue of WT soybean cultivar Enrei was used for qRT-PCR. **(B)** Total RNA from primary leaves of WT soybean cultivar Enrei during dark treatment was used for qRT-PCR. Expression levels were standardized using *GmACTIN*. Error bars indicate the standard error (SE; n = 5 biological replicates).

Among the various tissues of Enrei, *ELS1*/*GmBCM1* was highly expressed in mature leaves, and its expression level in different tissues was similar to that in *GmBCM2* (Glyma.01G198500, classical locus *G*), *GmBCM1* paralog (Figure 3A). Since *Arabidopsis BCM1* is induced by light and *BCM2* is induced by leaf senescence, we investigated the expression pattern of *ELS1*/*GmBCM1* and *GmBCM2* in soybean during dark treatment. The expression levels of both *ELS1*/*GmBCM1* and *GmBCM2* rapidly decreased at 1 DAD and remained low after 2 DAD (Figure 3B). Expression of the antenna protein genes *GmLhca1* and *GmLhcb1* is induced by light, and that of the chlorophyll-degrading enzyme genes *GmSGR1* and *GmNYC1* are influenced by leaf senescence (Figure 3B). Taken together, *ELS1*/*GmBCM1* and *GmBCM2,* unlike *Arabidopsis BCM2,* are genes induced by light and not by leaf senescence.

### Physiological Changes of Photosynthetic Proteins in *els1-2*

**Figure 4:**
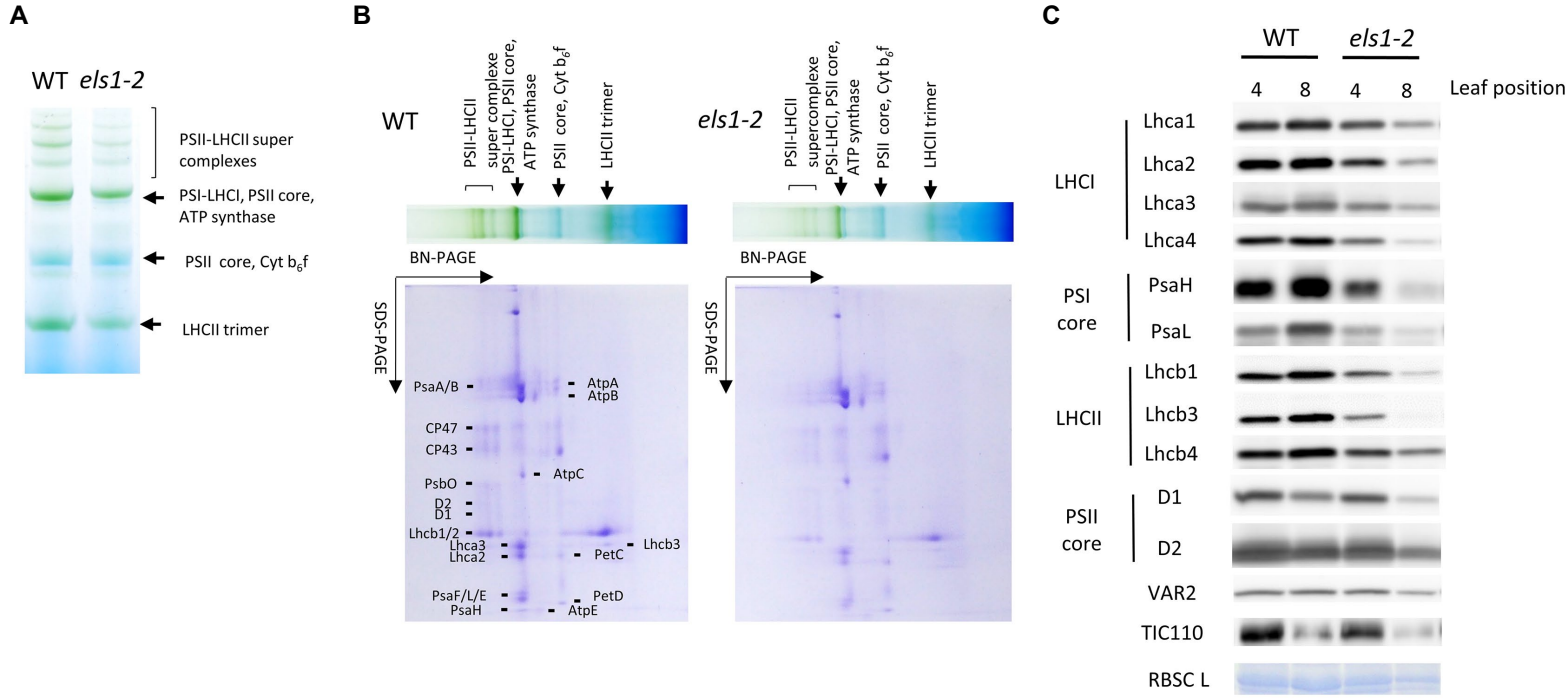
Thylakoid protein accumulation in *els1-2*. To examine the thylakoid membrane proteins and pigment complexes from pre-senescent leaves (the upper 4th leaves) of WT and *els1-2*, we performed **(A)** blue native PAGE analysis and **(B)** two-dimensional electrophoresis. SDS-PAGE was visualized by CBB staining. **(C)** Western blot of chloroplast proteins from pre-senescent (the upper 4th leaves) and senescent (the upper 8th leaves) leaves of WT and *els1-2*. Lhca1-4 are LHCI apoproteins; PsaH and L are photosystem I reaction center subunits; Lhcb1, 3, and 4 are LHCII apoproteins; D1 and D2 are photosystem II reaction center subunits; VAR2, TIC110, and RBSC L represent thylakoid membrane-localized protein, envelope-localized translocon protein, and stroma-localized protein, respectively. RBSC L was visualized by CBB staining.

As *els1-2* reveals a pale green phenotype at 0 WAF (SPAD value of the upper 4th pre-senescent leaves: WT, 36.65 ± 0.73; *els1-2*, 27.95 ± 0.91, *p* < 0.01), the composition of the photosystem proteins may have changed. The accumulation of PSII-LHCII super-complex and LHCII trimer in pre-senescent leaves of *els1-2* was lower than in the WT (Figures 4A,B). The composition of chloroplast proteins of D1 and D2 of the PSII core subunits in pre-senescent leaves did not differ between the WT and *els1-2* (Figure 4C). In contrast, the amount of PSI core subunits PsaH and PsaL, LHCI subunits Lhca1-4 and LHCII subunits Lhcb1, Lhcb3, and Lhcb4 of *els1-2* was lower than that of WT. The reduced amount of Chl *b*-binding protein of LHCI and LHCII in pre-senescent leaves in *els1-2* was consistent with increment of the Chl *a*/*b* ratio as mentioned above (Supplemental Figure 1). The chloroplast proteins that are not chlorophyll-binding proteins, YELLOW VARIEGATED2 (VAR2), translocon at the inner envelope membrane of chloroplasts 110 (TIC110), and Rubisco large subunit (RBSCL), were not different between the WT and *els1-2* in pre-senescent leaves. These results indicate that the loss of function of ELS1 altered the composition of the chlorophyll-binding proteins PSI, LHCI and LHCII in pre-senescent leaves.

In senescent leaves (the upper 8th leaves) at 0 WAF (SPAD value: WT, 34.80 ± 1.04; *els1-2*, 12.88 ± 1.12, *p* < 0.01), the amount of PSI, PSII, LHCI and LHCII of *els1-2* was reduced earlier than that of WT. In senescent leaves, the accumulation of RBSCLs in *els1-2* was reduced faster than in the WT, consistent with the physiological loss of leaf function in *els1-2* as described above.

The expression levels of *Lhca1*, *Lhca2, Lhcb1*, *Lhcb2*, *PsaH*, and *PsaL* in pre-senescent leaves of *els1-2* were slightly higher than those in the WT, but the difference was not significant (Supplemental Figure 5). These results indicate that the decreased accumulation of PSI core protein and LHC in *els1* was not due to reduced gene expression.

### Comparison of Agricultural Traits of Two Different Mutant Alleles

**Figure 5:**
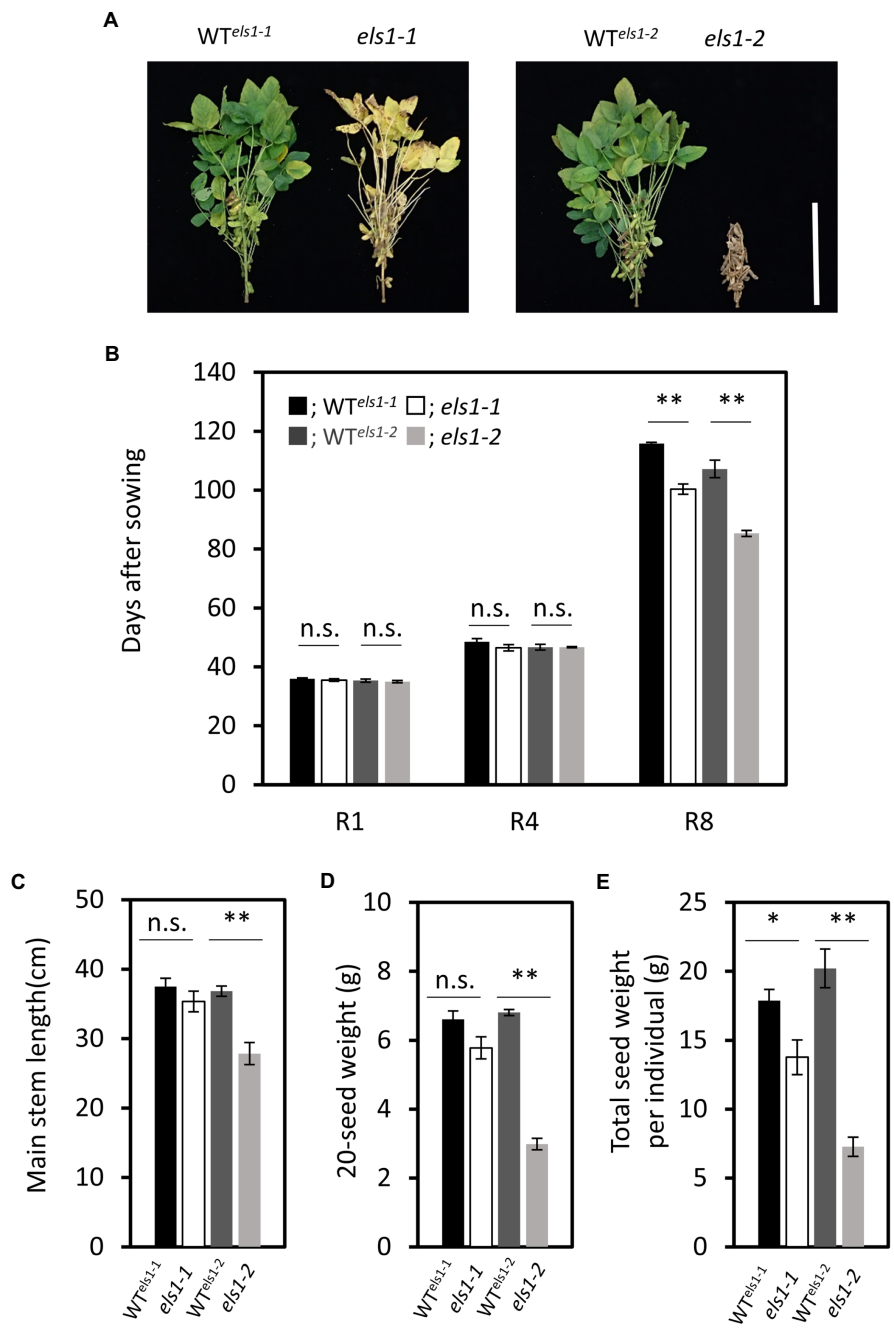
The differences of agronomic traits between *els1* and their WT. **(A)** Phenotype of WT *^els1-1^*, *els1-1*, WT *^els1-2^* and *els1-2* at about 80 days after sowing. The scale bar indicates 30 cm. **(B)** Days to each growth stage in *els1* (n = 6 biological replicates). Black bars indicate WT of *els1-1*, white bars indicate *els1-1*. Dark gray bars indicate WT of *els1-2*, light gray bars indicate *els1-2*. Error bars indicate the standard error (SE). *p < 0.05, **p < 0.01, n.s., not significant (Student’s t-test). **(C)** Main stem length in *els1* (n = 6 biological replicates). **(D)** 20-seed weight of *els1* (n = 6 biological replicates). **(E)** The total seed weight per plant in *els1* (n = 6 biological replicates).

The *els1* showed an early maturation phenotype than the WTs, especially *els1-2* matured earlier relative to the *els1-1* (Figure 5A). To examine in more detail, the number of days to reach each growth stage from R1 to R8 of *els1-1* and *els1-2* was compared with those of their WTs. The counterparts of WTs for *els1-1* and *els1-2* in the segregating population were named WT*^els1-1^* and WT*^els1-2^*, respectively. The flowering initiation stage R1 and the pod elongation stage R4 of *els1-1* and *els1-2* were not significantly different from those in WTs (days to each stage, R1: WT*^els1-1^*, 36.00 ± 0.26 day; *els1-1*, 35.50 ± 0.43 day; WT*^els1-2^*, 35.33 ± 0.49 day; *els1-2*, 35.00 ± 0.37 day; R4: WT*^els1-1^*, 48.50 ± 1.15 day; *els1-1*, 46.50 ± 1.06 day; WT*^els1-2^*, 46.67 ± 0.99 day; *els1-2*, 46.67 ± 0.21 day; Figure 5B). In contrast, the days to full ripening stage R8 in *els1-1* and *els1-2* were significantly earlier than those of the WT (R8: WT*^els1-1^*, 115.83 ± 0.40 day; *els1-1*, 100.33 ± 1.74 day; WT*^els1-2^*, 107.17 ± 2.97 day; *els1-2*, 85.33 ± 1.02 day; Figure 5B).

The main stem length of *els1-2* was significantly shorter than that of the WT (WT*^els1-2^*, 36.83 ± 0.75 cm; *els1-2*, 27.83 ± 1.60 cm) while that of *els1-1* was slightly shorter than that of the WT, but there was no significant difference (WT*^els1-1^*, 37.50 ± 1.20 cm; *els1-1*, 35.33 ± 1.48 cm; Figure 5C). The 20-seed weight of *els1-2* was significantly lower than that of the WT (WT*^els1-2^*, 6.81 ± 0.09 g; *els1-2*, 2.99 ± 0.17 g) while that of *els1-1* was slightly lower than that of the WT, but there was no significant difference (WT*^els1-1^*, 6.61 ± 0.24 g; *els1-1*, 5.78 ± 0.32 g; Figure 5D). Total seed weight per individual of *els1-2* was significantly lower than that of the WT (WT*^els1-2^*, 20.21 ± 1.40 g; *els1-2*, 7.27 ± 0.70 g) while that of *els1-1* was lower than the WT (WT*^els1-1^*, 17.87 ± 0.82 g; *els1-1*, 13.77 ± 1.25 g) and intermediate between WT and *els1-2* (Figure 5E).

## Discussion

### GmBCM1 Controls Chlorophyll Synthesis and Degradation in Higher Plants

In recent years, the causative genes of mutants and QTLs have been efficiently and quickly isolated using NGS analysis. The MutMap method developed in rice (Abe et al., 2012) was subsequently applied to soybean to characterize the causative genes of several mutants. Recently, the causative gene of the *spl-1* mutant induced by EMS was isolated by NGS-based bulk segregant analysis (Al Amin et al., 2019). NGS analysis was performed using 20 WT and 20 mutant bulks and they were narrowed down to seven candidate genes. Similarly, in the present study, we also efficiently narrowed down to one candidate gene of *els1-2* by NGS-based bulked segregant analysis and succeeded in identifying the causative genes of both *els1-1* and *els1-2* by genetic analysis (Figure 2).

Bulk DNA analysis using NGS revealed that *ELS1* encodes the ortholog of *Arabidopsis* BCM1, a chloroplast-localized CaaX-like protease (Figure 2). Other CaaX proteases such as Rce1 and Ste24 localize to the ER membrane (Bracha-Drori et al., 2008), regulate membrane localization of substrates, and clogging of the ER translocon (Ast et al., 2016). It has been reported that BCM1 localized in chloroplasts does not have CaaX protease activity and interacts with GUN4 and SGR to control chlorophyll synthesis and degradation (Wang et al., 2020). The *bcm1bcm2* double mutant in *Arabidopsis* exhibits phenotypes of pale green and early leaf senescence (Wang et al., 2020; Zhang et al., 2020). Similarly, *els1* revealed the phenotypes of pale green and early leaf senescence (Figure 1; Supplemental Figure 2), suggesting that its function is conserved among higher plants. Since soybean experienced two whole-genome duplications about 59 million years ago and 13 million years ago and has a paleopolyploid genome (Schmutz et al., 2010), nearly 75% of the genes are present in multiple copies. There are two copies of *GmBCM, GmBCM1,* and *GmBCM2*, in the soybean genome. *GmBCM2*, the paralog of *ELS1*/*GmBCM1*, has been reported as the causative gene that controls the green seed coat in soybean (Wang et al., 2018). The *GmBCM2* allele in the wild species (*G. soja*) is the function allele, whereas the loss-of-function mutant allele, *gmbcm2*, is common in cultivated soybean. Cultivated soybeans with yellow seed coats are caused by a loss of *GmBCM2* function, resulting in decreased chlorophyll synthesis and/or increased chlorophyll degradation. The *GmBCM2* allele of the WT donor plant of the mutant library is *gmbcm2*; therefore, *els1-1* and *els1-2* are the *gmbcm1gmbcm2* double mutants. Duplicated genes may undergo pseudogenization, sub-functionalization, or neofunctionalization (Innan and Kondrashov, 2010). Since cultivated yellow soybeans with the *gmbcm2* mutation do not reduce green leaf color, the two GmBCMs are likely working redundantly in soybean leaves. The strong expression levels of both *GmBCM1* and *GmBCM2* in the leaves support this hypothesis (Figure 3A). Phenotypes of pale green and early chlorophyll degradation in *els1* were more pronounced due to the loss of both *GmBCM* gene functions.

In *Arabidopsis*, *BCM1* expression is induced by light, whereas *BCM2* expression is induced by leaf senescence (Wang et al., 2020; Zhang et al., 2020). Interestingly, *ELS1*/*GmBCM1* and *GmBCM2* expression in soybean rapidly decrease at 1DAD, suggesting that both GmBCMs regulate light responses during photosynthesis (Figure 3B). Since many genes responsible for the light reaction during photosynthesis are strongly expressed in green tissues such as leaves and are induced by light, these findings were consistent with *GmBCM1* and *GmBCM2*. In contrast, *ELS1*/*GmBCM1* and *GmBCM2* expression were not induced by dark-induced leaf senescence at 6DAD, suggesting that their expression regulation might differ from *Arabidopsis BCM2* (Figure 3B).

Blue native PAGE analysis and western blot analyses showed that the amount of PSI, LHCI and LHCII was reduced in *els1-2* compared to the WT in the pre-senescent leaves (Figure 4). In contrast, the accumulation of PSII core protein and other chloroplast-localized proteins was not notably different between the WT and *els1-2*. The Chl *a*/*b* ratio of *els1-2* was higher in the pre-senescent leaves than in the WT (Supplemental Figure 1). This was consistent with the protein analysis that the Chl *a/b* ratio increased with decreasing accumulation of LHC (the only Chl *b* binding protein). Thus, the pale green phenotype in *els1* could be due to the reduced amounts of PSI and LHC (Figure 4). The expression levels of PSI and LHC genes were not reduced in *els1-2* as compared with the WT (Supplemental Figure 5). These results suggest that ELS1 regulates the light reaction of photosynthesis by controlling the accumulation of PSI and LHC at the protein level.

BCM1 in *Arabidopsis* destabilizes the magnesium dechelatase SGR, and the *bcm1bcm2* double mutant accumulates SGR even before leaf senescence and shows an early chlorophyll degradation phenotype (Wang et al., 2020). An early leaf senescence phenotype in *els1* might be due to the high accumulation of SGR, similar to the *Arabidopsis bcm1bcm2* double mutant. Since there was no significant difference in *SGR* expression before leaf senescence between the WT and *els1-2*, the regulation of SGR accumulation by BCM is likely to occur at the protein level in soybean (Figure 1D; Supplemental Figure 2C). In senescent leaves of *els1-2*, the amount of PSI and LHC subunits decreased faster, and the Chl *a*/*b* ratio gradually increased (Figure 4). Previous studies have proposed that the degradation of PSI and LHC is regulated by SGR-mediated chlorophyll degradation, and the degradation of PS II is regulated by NYC4/THF1 (Huang et al., 2013; Yamatani et al., 2013). BCM1 inhibits chlorophyll degradation *via* SGR, and the pronounced degradation of PSI and LHC in senescent leaves of *els1-2* was consistent with previous results. In this study, *els1* showed early chlorophyll degradation during leaf senescence and decreased carbon assimilation rate and induction of senescence-related genes, indicating an early leaf senescence phenotype (Figure 1D; Supplemental Figure 2C). The *Arabidopsis bcm1bcm2* double mutant exhibited premature chlorophyll degradation during senescence, but decreased leaf functionality was similar to that of the WT (Wang et al., 2020). In contrast, *SGR* overexpression lines showed early chlorophyll degradation and decreased leaf functionality (Park et al., 2007). Since BCM1 destabilizes SGR and inhibits chlorophyll degradation, we speculated that reduced leaf functionality in *els1* might occur after early chlorophyll degradation due to the high accumulation of SGR.

### *els1* Accelerates Maturation by Early Chlorophyll Degradation

In this study, we successfully isolated two *els1* alleles. Mutations in *els1-1* and *els1-2* caused the conserved amino acid substitution from serine to proline at position 233aa and nonsense mutation at position 349aa, respectively (Figure 2). The position of the amino acid substitution in *els1-1* is conserved between *Arabidopsis* and rice (Wang et al., 2018). Although there are no reports on the functional domain of the region, this amino acid residue is expected to be important for the function of the GmBCM1 protein because *els1-1* showed a pale green and early senescence phenotype. Both *els1-1* and *els1-2* mutants revealed a pale green phenotype and early leaf senescence, but the extent differed (Figure 1B). These results suggest that *els1-1* is a weak allele with impaired protein function, whereas *els1-2* is a strong allele with complete loss of function. Recently, natural variations of *gmbcm1*/*yl2* and *gmgcm2*/*yl1* have been isolated (Liu et al., 2020). The double mutant of *yl1yl2* exhibited phenotypes of pale green and early chlorophyll degradation, similar to *els1*. *yl1* has been reported to have a 1 bp deletion resulting in a frameshift and null mutation in *GmBCM2*. Although the effect of *yl1* variation on agricultural traits has not been reported yet, there may be a significant effect due to early chlorophyll degradation, such as *els1-2*.

The days to flowering (R1) and pod elongation (R4) of *els1-1* and *els1-2* were almost the same as those of the WT. On the other hand, the days to the full ripening of pod (R8) of *els1-1* and *els1-2* were significantly less than those of the WT (Figure 5B). For example, the number of days until the full ripening stage (R8) of *els1-2* was 20 days shorter than the WT. The plant height of *els1-1* was slightly lower than the WT, but the difference was not significant (Figure 5C). In addition, the plant height of *els1-1* decreased mildly compared to that of *els1-2*. The total seed weight per individual of *els1-1* was slightly lower than that of the WT, but its extent was intermediate between the WT and *els1-2* (Figure 5D). In other words, the weak allele of *els1-1* is expected to accelerate the harvest time without significantly affecting agronomic traits, such as flowering time and yield.

Moreover, early chlorophyll degradation in *els1* is expected to suppress green stem disorders. Interestingly, the heterozygous line between *els1-1* and *els1-2* showed an intermediate chlorophyll degradation phenotype (Figure 1B). This indicates that the phenotype of *els1* might be able to quantitatively manipulate early chlorophyll degradation depending on the strength of the allele. Isolation of novel alleles of different strengths of *ELS1* might allow for fine-tuning of the harvesting period without affecting agronomic traits.

## Data Availability Statement

The datasets presented in this study can be found in online repositories. The names of the repository/repositories and accession number(s) can be found in the article/Supplementary Material.

## Author Contributions

HY and AK designed the study. HY, TH, and AK conducted the study. TY and MK provided information and advice regarding gene function analysis. HY, TY, MK, and AK wrote the manuscript. All authors contributed to the article and approved the submitted version.

## Funding

This study was supported by JSPS KAKENHI (Grant Number 19 J00195) to HY and a grant from the Ministry of Agriculture, Forestry, and Fisheries of Japan [Genomics-Based Technology for Agricultural Improvement (IVG1005)] to AK.

## Conflict of Interest

The authors declare that the research was conducted without any commercial or financial relationships that could be construed as a potential conflict of interest.

## Publisher’s Note

All claims expressed in this article are solely those of the authors and do not necessarily represent those of their affiliated organizations, or those of the publisher, the editors and the reviewers. Any product that may be evaluated in this article, or claim that may be made by its manufacturer, is not guaranteed or endorsed by the publisher.

## Abbreviations

Chl *a*: Chlorophyll *a*
Chl *b*: Chlorophyll *b*
DAD: Days after dark incubation
*els1*: Early leaf senescence 1
FW: Fresh weight
GSD: Green stem disorder
LHCI: Light-harvesting complex II
LHCII: Light-harvesting complex II
NGS: Next-generation sequencing
PSI: Photosystem I
PSII: Photosystem II
qRT-PCR: Quantitative RT-PCR
SNP: Single nucleotide polymorphism
WAH: Weeks after flowering
WT: Wild-type.

## References

[ref1] Abe A. Kosugi S. Yoshida K. Natsume S. Takagi H. Kanzaki H.. (2012). Genome sequencing reveals agronomically important loci in rice using MutMap. Nat. Biotechnol. 30, 174–178. doi: 10.1038/nbt.2095, PMID: 22267009

[ref2] Al Amin G. M. Kong K. Sharmin R. A. Kong J. Bhat J. A. Zhao T. (2019). Characterization and rapid gene-mapping of leaf lesion mimic phenotype of *spl-1* mutant in soybean (*Glycine max* (L.) merr.). Int. J. Mol. Sci. 20, 2193. doi: 10.3390/ijms20092193, PMID: 31058828 PMC6539437

[ref3] Apel K. Hirt H. (2004). Reactive oxygen species: metabolism, oxidative stress, and signal transduction. Annu. Rev. Plant Biol. 55, 373–399. doi: 10.1146/annurev.arplant.55.031903.141701 15377225

[ref4] Armstead I. Donnison I. Aubry S. Harper J. Hörtensteiner S. James C.. (2007). Cross-species identification of Mendel’s *I* locus. Science 315, 73. doi: 10.1126/science.1132912 17204643

[ref5] Ast T. Michaelis S. Schuldiner M. (2016). The protease Ste24 clears clogged translocons. Cell 164, 103–114. doi: 10.1016/j.cell.2015.11.053, PMID: 26771486 PMC4715265

[ref6] Bracha-Drori K. Shichrur K. Lubetzky T. C. Yalovsky S. (2008). Functional analysis of *Arabidopsis* postprenylation CaaX processing enzymes and their function in subcellular protein targeting. Plant Physiol. 148, 119–131. doi: 10.1104/pp.108.120477, PMID: 18641086 PMC2528099

[ref7] Davison P. A. Schubert H. L. Reid J. D. Iorg C. D. Heroux A. Hill C. P.. (2005). Structural and biochemical characterization of Gun4 suggests a mechanism for its role in chlorophyll biosynthesis. Biochemistry 44, 7603–7612. doi: 10.1021/bi050240x, PMID: 15909975

[ref8] Dougherty L. Singh R. Brown S. Dardick C. Xu K. (2018). Exploring DNA variant segregation types in pooled genome sequencing enables effective mapping of weeping trait in *Malus*. J. Exp. Bot. 69, 1499–1516. doi: 10.1093/jxb/erx490, PMID: 29361034 PMC5888915

[ref9] Fehr W. R. Caviness C. E. Burmood D. T. Pennington J. S. (1971). Stage of development descriptions for soybeans, *glycine max* (L.) Merrill. Crop Sci. 11, 929–931. doi: 10.2135/cropsci1971.0011183X001100060051x

[ref10] Gibson L. C. D. Willows R. D. Kannangara C. G. von Wettstein D. Hunter C. N. (1995). Magnesium-protoporphyrin chelatase of *Rhodobacter sphaeroides*: reconstitution of activity by combining the products of the *bchH*, *-I*, and *-D* genes expressed in *Escherichia coli*. Proc. Natl. Acad. Sci. 92, 1941–1944. doi: 10.1073/pnas.92.6.1941, PMID: 7892204 PMC42398

[ref11] Hill C. B. Hartman G. L. Esgar R. Hobbs H. A. (2006). Field evaluation of green stem disorder in soybean cultivars. Crop Sci. 46, 879–885. doi: 10.2135/cropsci2005.0207

[ref12] Hobbs H. A. Hill C. B. Grau C. R. Koval N. C. Wang Y. Pedersen W. L.. (2006). Green stem disorder of soybean. Plant Dis. 90, 513–518. doi: 10.1094/PD-90-0513, PMID: 30786603

[ref13] Huang W. Chen Q. Zhu Y. Hu F. Zhang L. Ma Z.. (2013). *Arabidopsis* thylakoid formation 1 is a critical regulator for dynamics of PSII-LHCII complexes in leaf senescence and excess light. Mol. Plant 6, 1673–1691. doi: 10.1093/mp/sst069, PMID: 23671330

[ref14] Innan H. Kondrashov F. (2010). The evolution of gene duplications: classifying and distinguishing between models. Nat. Rev. Genet. 11, 97–108. doi: 10.1038/nrg2689, PMID: 20051986

[ref15] Kato Y. Sun X. Zhang L. Sakamoto W. (2012). Cooperative D1 degradation in the photosystem II repair mediated by chloroplastic proteases in Arabidopsis. Plant Physiol. 159, 1428–1439. doi: 10.1104/pp.112.199042, PMID: 22698923 PMC3425188

[ref16] Kikuchi S. Bédard J. Hirano M. Hirabayashi Y. Oishi M. Imai M.. (2013). Uncovering the protein translocon at the chloroplast inner envelope membrane. Science 339, 571–574. doi: 10.1126/science.1229262, PMID: 23372012

[ref17] Kohzuma K. Sato Y. Ito H. Okuzaki A. Watanabe M. Kobayashi H.. (2017). The non-mendelian green cotyledon gene in soybean encodes a small subunit of photosystem II. Plant Physiol. 173, 2138–2147. doi: 10.1104/pp.16.01589, PMID: 28235890 PMC5373049

[ref18] Kusaba M. Ito H. Morita R. Iida S. Sato Y. Fujimoto M.. (2007). Rice NON-YELLOW COLORING1 is involved in light-harvesting complex II and grana degradation during leaf senescence. Plant Cell 19, 1362–1375. doi: 10.1105/tpc.106.042911, PMID: 17416733 PMC1913755

[ref19] Larkin R. M. Alonso J. M. Ecker J. R. Chory J. (2003). GUN4, a regulator of chlorophyll synthesis and intracellular signaling. Science 299, 902–906. doi: 10.1126/science.1079978, PMID: 12574634

[ref20] Liu B. Kanazawa A. Matsumura H. Takahashi R. Harada K. Abe J. (2008). Genetic redundancy in soybean photoresponses associated with duplication of the phytochrome A gene. Genetics 180, 995–1007. doi: 10.1534/genetics.108.092742, PMID: 18780733 PMC2567397

[ref21] Liu M. Wang Y. Nie Z. Gai J. Bhat J. A. Kong J.. (2020). Double mutation of two homologous genes *YL1* and *YL2* results in a leaf yellowing phenotype in soybean [*Glycine max* (L.) Merr]. Plant Mol. Biol. 103, 527–543. doi: 10.1007/s11103-020-01008-9, PMID: 32323129

[ref22] Lu S. Dong L. Fang C. Liu S. Kong L. Cheng Q.. (2020). Stepwise selection on homeologous *PRR* genes controlling flowering and maturity during soybean domestication. Nat. Genet. 52, 428–436. doi: 10.1038/s41588-020-0604-7, PMID: 32231277

[ref23] Ono K. Kimura M. Matsuura H. Tanaka A. Ito H. (2019). Jasmonate production through chlorophyll *a* degradation by stay-green in *Arabidopsis thaliana*. J. Plant Physiol. 238, 53–62. doi: 10.1016/j.jplph.2019.05.004, PMID: 31136906

[ref24] Park S. Y. Yu J. W. Park J. S. Li J. Yoo S. C. Lee N. Y.. (2007). The senescence-induced staygreen protein regulates chlorophyll degradation. Plant Cell 19, 1649–1664. doi: 10.1105/tpc.106.044891, PMID: 17513504 PMC1913741

[ref25] Phillips D. A. Pierce R. O. Edie S. A. Foster K. A. Knowles P. F. (1984). Delayed leaf senescence in soybean. Crop Sci. 24, 518–522. doi: 10.2135/cropsci1984.0011183X002400030022x

[ref26] Porra R. J. Thompson W. A. Kriedemann P. E. (1989). Determination of accurate extinction coefficients and simultaneous equations for assaying chlorophylls *a* and *b* extracted with four different solvents: verification of the concentration of chlorophyll standards by atomic absorption spectroscopy. Biochim. Biophys. Acta Bioenerg. 975, 384–394. doi: 10.1016/S0005-2728(89)80347-0

[ref27] Ren G. An K. Liao Y. Zhou X. Cao Y. Zhao H.. (2007). Identification of a novel chloroplast protein AtNYE1 regulating chlorophyll degradation during leaf senescence in Arabidopsis. Plant Physiol. 144, 1429–1441. doi: 10.1104/pp.107.100172, PMID: 17468209 PMC1914121

[ref28] Sakamoto W. Zaltsman A. Adam Z. Takahashi Y. (2003). Coordinated regulation and complex formation of YELLOW VARIEGATED1 and YELLOW VARIEGATED2, chloroplastic FtsH metalloproteases involved in the repair cycle of photosystem II in Arabidopsis thylakoid membranes. Plant Cell 15, 2843–2855. doi: 10.1105/tpc.017319, PMID: 14630971 PMC282813

[ref29] Sato Y. Morita R. Katsuma S. Nishimura M. Tanaka A. Kusaba M. (2009). Two short-chain dehydrogenase/reductases, NON-YELLOW COLORING 1 and NYC1-LIKE, are required for chlorophyll *b* and light-harvesting complex II degradation during senescence in rice. Plant J. 57, 120–131. doi: 10.1111/j.1365-313X.2008.03670.x, PMID: 18778405

[ref30] Sato Y. Morita R. Nishimura M. Yamaguchi H. Kusaba M. (2007). Mendel’s green cotyledon gene encodes a positive regulator of the chlorophyll-degrading pathway. Proc. Natl. Acad. Sci. 104, 14169–14174. doi: 10.1073/pnas.0705521104, PMID: 17709752 PMC1955798

[ref31] Schmutz J. Cannon S. B. Schlueter J. Ma J. Mitros T. Nelson W.. (2010). Genome sequence of the palaeopolyploid soybean. Nature 463, 178–183. doi: 10.1038/nature08670, PMID: 20075913

[ref32] Shimoda Y. Ito H. Tanaka A. (2016). Arabidopsis *STAY-GREEN*, Mendel’s green cotyledon gene, encodes magnesium-dechelatase. Plant Cell 28, 2147–2160. doi: 10.1105/tpc.16.00428, PMID: 27604697 PMC5059807

[ref33] Tanaka A. Ito H. Tanaka R. Tanaka N. K. Yoshida K. Okada K. (1998). Chlorophyll *a* oxygenase (*CAO*) is involved in chlorophyll *b* formation from chlorophyll *a*. Proc. Natl. Acad. Sci. 95, 12719–12723. doi: 10.1073/pnas.95.21.12719, PMID: 9770552 PMC22897

[ref34] Tanaka Y. Taniyoshi K. Imamura A. Mukai R. Sukemura S. Sakoda K.. (2021). MIC-100, a new system for high-throughput phenotyping of instantaneous leaf photosynthetic rate in the field. Funct. Plant Biol. doi: 10.1071/FP21029, PMID: 34090541

[ref35] Tsuda M. Kaga A. Anai T. Shimizu T. Sayama T. Takagi K.. (2015). Construction of a high-density mutant library in soybean and development of a mutant retrieval method using amplicon sequencing. BMC Genomics 16, 1014. doi: 10.1186/s12864-015-2079-y, PMID: 26610706 PMC4662035

[ref36] Verdecia M. A. Larkin R. M. Ferrer J.-L. Riek R. Chory J. Noel J. P. (2005). Structure of the mg-chelatase cofactor GUN4 reveals a novel hand-shaped fold for porphyrin binding. PLoS Biol. 3:e151. doi: 10.1371/journal.pbio.0030151, PMID: 15884974 PMC1084334

[ref37] Wang M. Li W. Fang C. Xu F. Liu Y. Wang Z.. (2018). Parallel selection on a dormancy gene during domestication of crops from multiple families. Nat. Genet. 50, 1435–1441. doi: 10.1038/s41588-018-0229-2, PMID: 30250128

[ref38] Wang P. Richter A. S. Kleeberg J. R. W. Geimer S. Grimm B. (2020). Post-translational coordination of chlorophyll biosynthesis and breakdown by BCMs maintains chlorophyll homeostasis during leaf development. Nat. Commun. 11, 1254. doi: 10.1038/s41467-020-14992-9, PMID: 32198392 PMC7083845

[ref39] Watanabe S. Hideshima R. Xia Z. Tsubokura Y. Sato S. Nakamoto Y.. (2009). Map-based cloning of the gene associated with the soybean maturity locus *E3*. Genetics 182, 1251–1262. doi: 10.1534/genetics.108.098772, PMID: 19474204 PMC2728863

[ref40] Watanabe S. Xia Z. Hideshima R. Tsubokura Y. Sato S. Yamanaka N.. (2011). A map-based cloning strategy employing a residual heterozygous line reveals that the *GIGANTEA* gene is involved in soybean maturity and flowering. Genetics 188, 395–407. doi: 10.1534/genetics.110.125062, PMID: 21406680 PMC3122305

[ref41] Xia Z. Watanabe S. Yamada T. Tsubokura Y. Nakashima H. Zhai H.. (2012). Positional cloning and characterization reveal the molecular basis for soybean maturity locus *E1* that regulates photoperiodic flowering. Proc. Natl. Acad. Sci. 109, E2155–E2164. doi: 10.1073/pnas.1117982109, PMID: 22619331 PMC3420212

[ref42] Yamatani H. Kohzuma K. Nakano M. Takami T. Kato Y. Hayashi Y.. (2018). Impairment of Lhca4, a subunit of LHCI, causes high accumulation of chlorophyll and the stay-green phenotype in rice. J. Exp. Bot. 69, 1027–1035. doi: 10.1093/jxb/erx468, PMID: 29304198 PMC6019047

[ref43] Yamatani H. Sato Y. Masuda Y. Kato Y. Morita R. Fukunaga K.. (2013). *NYC4*, the rice ortholog of Arabidopsis *THF1*, is involved in the degradation of chlorophyll-protein complexes during leaf senescence. Plant J. 74, 652–662. doi: 10.1111/tpj.12154, PMID: 23432654

[ref44] Zhang C. Zhang B. Mu B. Zheng X. Zhao F. Lan W.. (2020). A thylakoid membrane protein functions synergistically with GUN5 in chlorophyll biosynthesis. Plant Commun. 1:100094. doi: 10.1016/j.xplc.2020.100094, PMID: 33367259 PMC7747962

