# Supplementary material for: Identification and Characterization of an Early Leaf Senescence Gene ELS1 in Soybean
Source: Front Plant Sci. 2021 Dec 16;12:784105. doi: 10.3389/fpls.2021.784105 (PMC8716371; doi:10.3389/fpls.2021.784105)
Supplement: Supplementary file 1 [file Presentation_1.PPTX]

## Slide 1
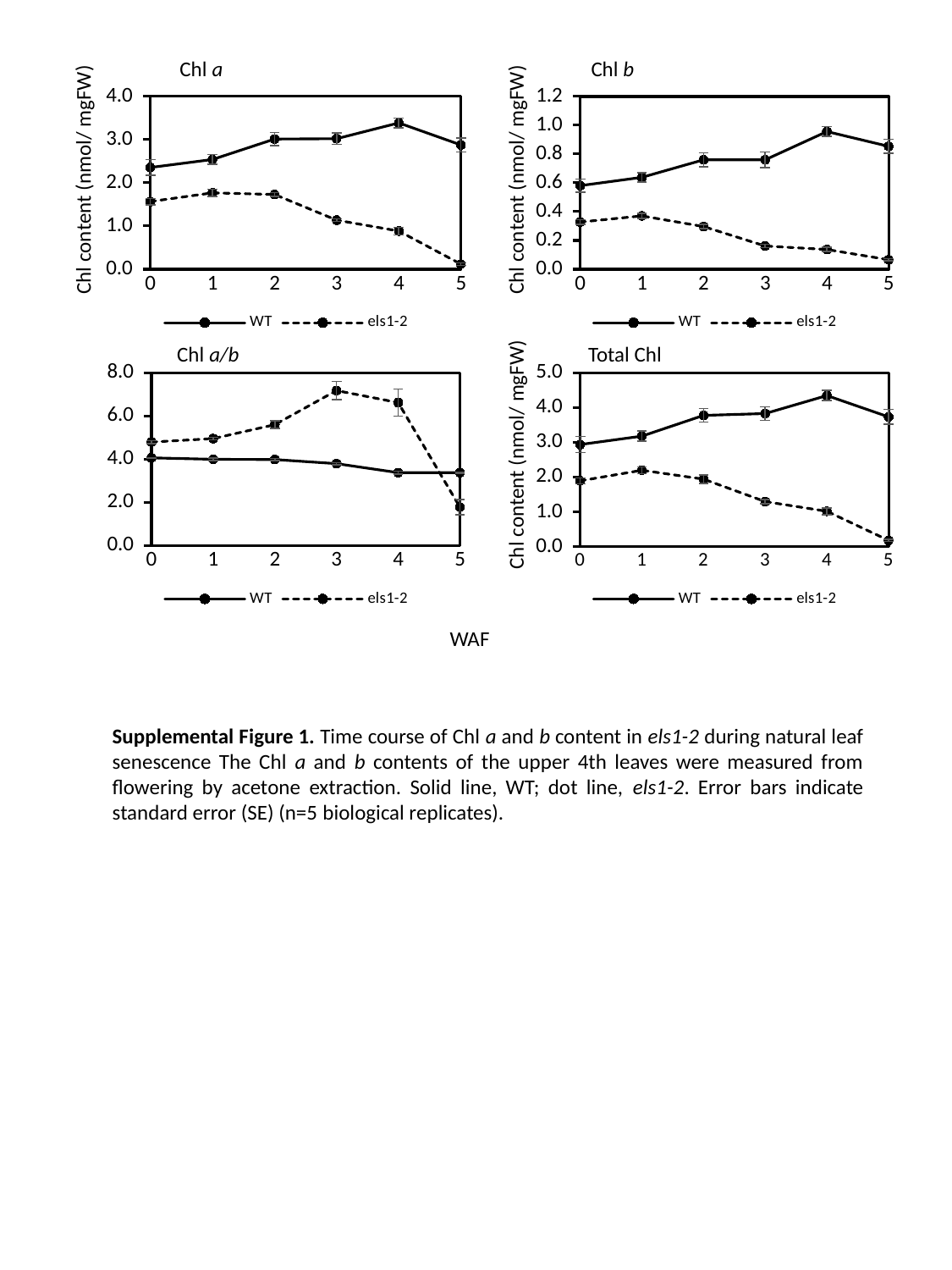

Chl b
Chl a
### Chart
| Category | WT | els1-2 |
|---|---|---|
### Chart
| Category | WT | els1-2 |
|---|---|---|Chl content (nmol/ mgFW)
Chl content (nmol/ mgFW)
Total Chl
Chl a/b
### Chart
| Category | WT | els1-2 |
|---|---|---|
### Chart
| Category | WT | els1-2 |
|---|---|---|Chl content (nmol/ mgFW)
WAF
Supplemental Figure 1. Time course of Chl a and b content in els1-2 during natural leaf senescence The Chl a and b contents of the upper 4th leaves were measured from flowering by acetone extraction. Solid line, WT; dot line, els1-2. Error bars indicate standard error (SE) (n=5 biological replicates).

## Slide 2
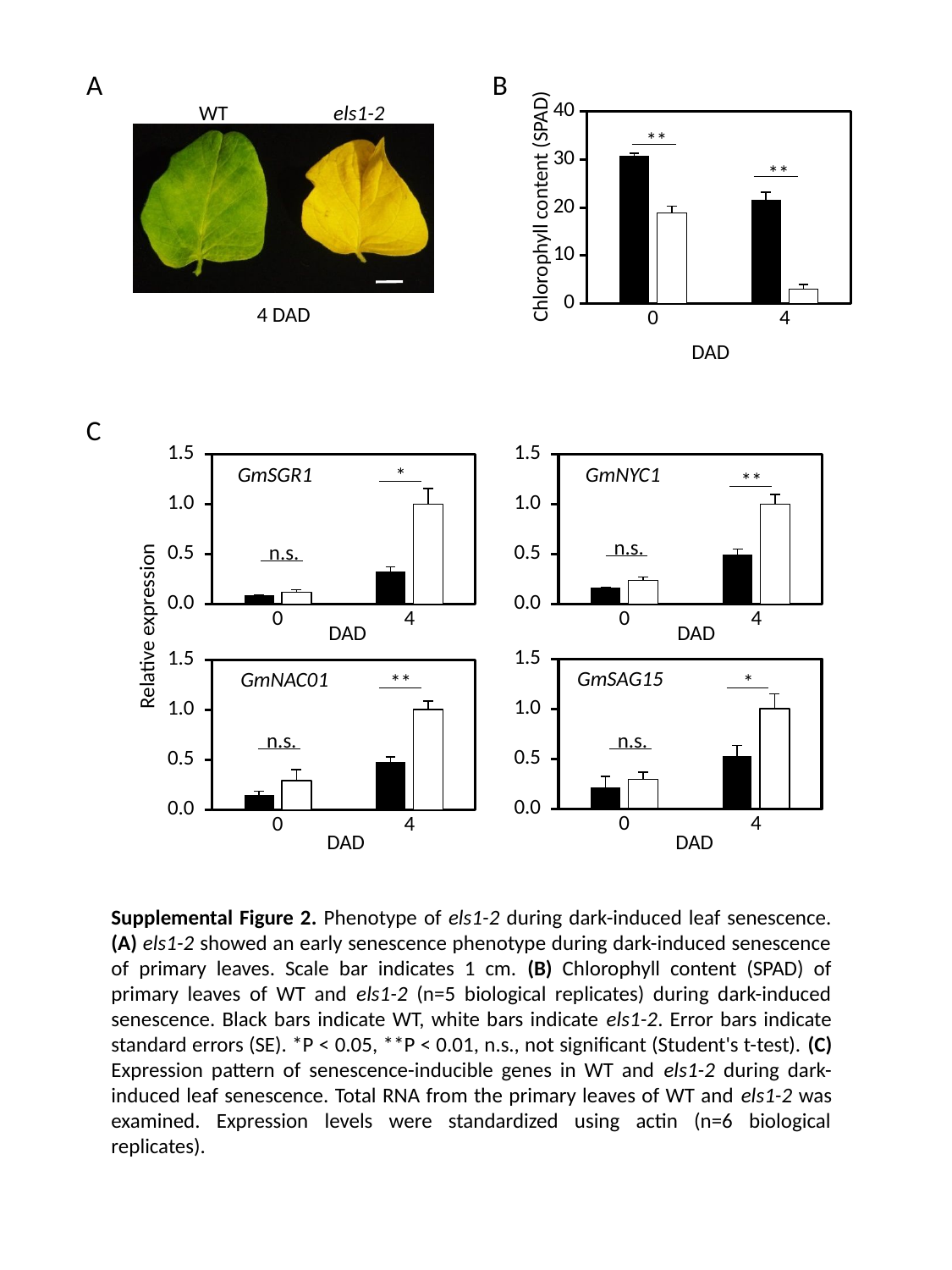

A
B
### Chart
| Category | WT | gslY349* |
|---|---|---|
| 0 | 30.74 | 18.939999999999998 |
| 4 | 21.6 | 2.94 |**
**
WT
els1-2
4 DAD
Chlorophyll content (SPAD)
DAD
C
### Chart
| Category | WT | gslY349* |
|---|---|---|
| 0 | 0.08656126701150416 | 0.11749896666892427 |
| 4 | 0.32835859339322926 | 1.0000222961967777 |
### Chart
| Category | WT | gslY349* |
|---|---|---|
| 0 | 0.160558579253763 | 0.2344142648119658 |
| 4 | 0.4950288953051506 | 1.000058481478578 |GmNYC1
GmSGR1
*
**
n.s.
n.s.
Relative expression
### Chart
| Category | WT | gslY349* |
|---|---|---|
| 0 | 0.21474689754291978 | 0.29296999821797415 |
| 4 | 0.5231989393447093 | 0.9999677628732732 |
### Chart
| Category | WT | gslY349* |
|---|---|---|
| 0 | 0.145775280658554 | 0.2895695158520585 |
| 4 | 0.4762036305553508 | 0.9999671432207213 |GmSAG15
GmNAC01
**
*
n.s.
n.s.
DAD
DAD
DAD
DAD
Supplemental Figure 2. Phenotype of els1-2 during dark-induced leaf senescence. (A) els1-2 showed an early senescence phenotype during dark-induced senescence of primary leaves. Scale bar indicates 1 cm. (B) Chlorophyll content (SPAD) of primary leaves of WT and els1-2 (n=5 biological replicates) during dark-induced senescence. Black bars indicate WT, white bars indicate els1-2. Error bars indicate standard errors (SE). *P < 0.05, **P < 0.01, n.s., not significant (Student's t-test). (C) Expression pattern of senescence-inducible genes in WT and els1-2 during dark-induced leaf senescence. Total RNA from the primary leaves of WT and els1-2 was examined. Expression levels were standardized using actin (n=6 biological replicates).

## Slide 3
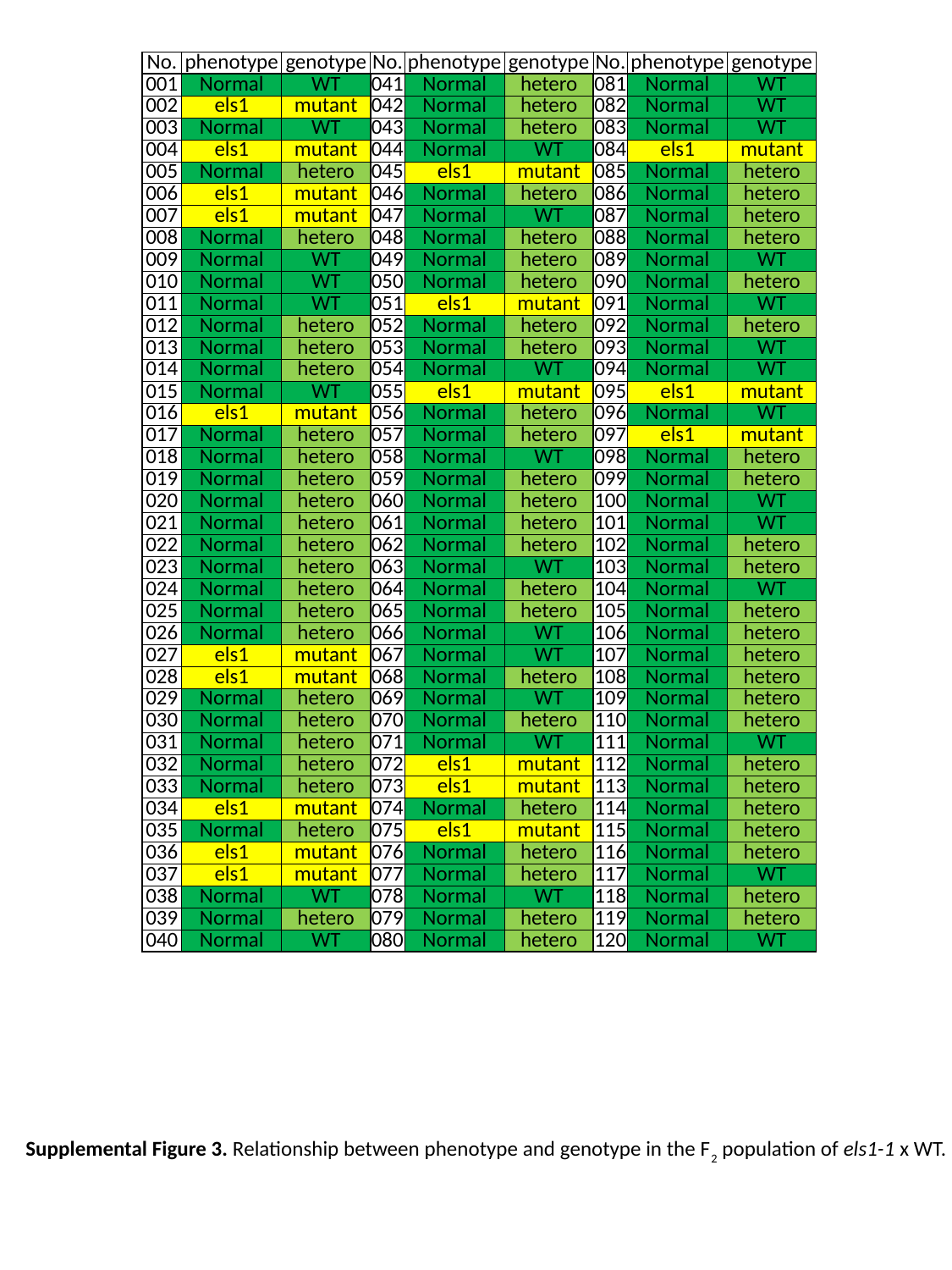

| No. | phenotype | genotype | No. | phenotype | genotype | No. | phenotype | genotype |
| --- | --- | --- | --- | --- | --- | --- | --- | --- |
| 001 | Normal | WT | 041 | Normal | hetero | 081 | Normal | WT |
| 002 | els1 | mutant | 042 | Normal | hetero | 082 | Normal | WT |
| 003 | Normal | WT | 043 | Normal | hetero | 083 | Normal | WT |
| 004 | els1 | mutant | 044 | Normal | WT | 084 | els1 | mutant |
| 005 | Normal | hetero | 045 | els1 | mutant | 085 | Normal | hetero |
| 006 | els1 | mutant | 046 | Normal | hetero | 086 | Normal | hetero |
| 007 | els1 | mutant | 047 | Normal | WT | 087 | Normal | hetero |
| 008 | Normal | hetero | 048 | Normal | hetero | 088 | Normal | hetero |
| 009 | Normal | WT | 049 | Normal | hetero | 089 | Normal | WT |
| 010 | Normal | WT | 050 | Normal | hetero | 090 | Normal | hetero |
| 011 | Normal | WT | 051 | els1 | mutant | 091 | Normal | WT |
| 012 | Normal | hetero | 052 | Normal | hetero | 092 | Normal | hetero |
| 013 | Normal | hetero | 053 | Normal | hetero | 093 | Normal | WT |
| 014 | Normal | hetero | 054 | Normal | WT | 094 | Normal | WT |
| 015 | Normal | WT | 055 | els1 | mutant | 095 | els1 | mutant |
| 016 | els1 | mutant | 056 | Normal | hetero | 096 | Normal | WT |
| 017 | Normal | hetero | 057 | Normal | hetero | 097 | els1 | mutant |
| 018 | Normal | hetero | 058 | Normal | WT | 098 | Normal | hetero |
| 019 | Normal | hetero | 059 | Normal | hetero | 099 | Normal | hetero |
| 020 | Normal | hetero | 060 | Normal | hetero | 100 | Normal | WT |
| 021 | Normal | hetero | 061 | Normal | hetero | 101 | Normal | WT |
| 022 | Normal | hetero | 062 | Normal | hetero | 102 | Normal | hetero |
| 023 | Normal | hetero | 063 | Normal | WT | 103 | Normal | hetero |
| 024 | Normal | hetero | 064 | Normal | hetero | 104 | Normal | WT |
| 025 | Normal | hetero | 065 | Normal | hetero | 105 | Normal | hetero |
| 026 | Normal | hetero | 066 | Normal | WT | 106 | Normal | hetero |
| 027 | els1 | mutant | 067 | Normal | WT | 107 | Normal | hetero |
| 028 | els1 | mutant | 068 | Normal | hetero | 108 | Normal | hetero |
| 029 | Normal | hetero | 069 | Normal | WT | 109 | Normal | hetero |
| 030 | Normal | hetero | 070 | Normal | hetero | 110 | Normal | hetero |
| 031 | Normal | hetero | 071 | Normal | WT | 111 | Normal | WT |
| 032 | Normal | hetero | 072 | els1 | mutant | 112 | Normal | hetero |
| 033 | Normal | hetero | 073 | els1 | mutant | 113 | Normal | hetero |
| 034 | els1 | mutant | 074 | Normal | hetero | 114 | Normal | hetero |
| 035 | Normal | hetero | 075 | els1 | mutant | 115 | Normal | hetero |
| 036 | els1 | mutant | 076 | Normal | hetero | 116 | Normal | hetero |
| 037 | els1 | mutant | 077 | Normal | hetero | 117 | Normal | WT |
| 038 | Normal | WT | 078 | Normal | WT | 118 | Normal | hetero |
| 039 | Normal | hetero | 079 | Normal | hetero | 119 | Normal | hetero |
| 040 | Normal | WT | 080 | Normal | hetero | 120 | Normal | WT |
Supplemental Figure 3. Relationship between phenotype and genotype in the F2 population of els1-1 x WT.

## Slide 4
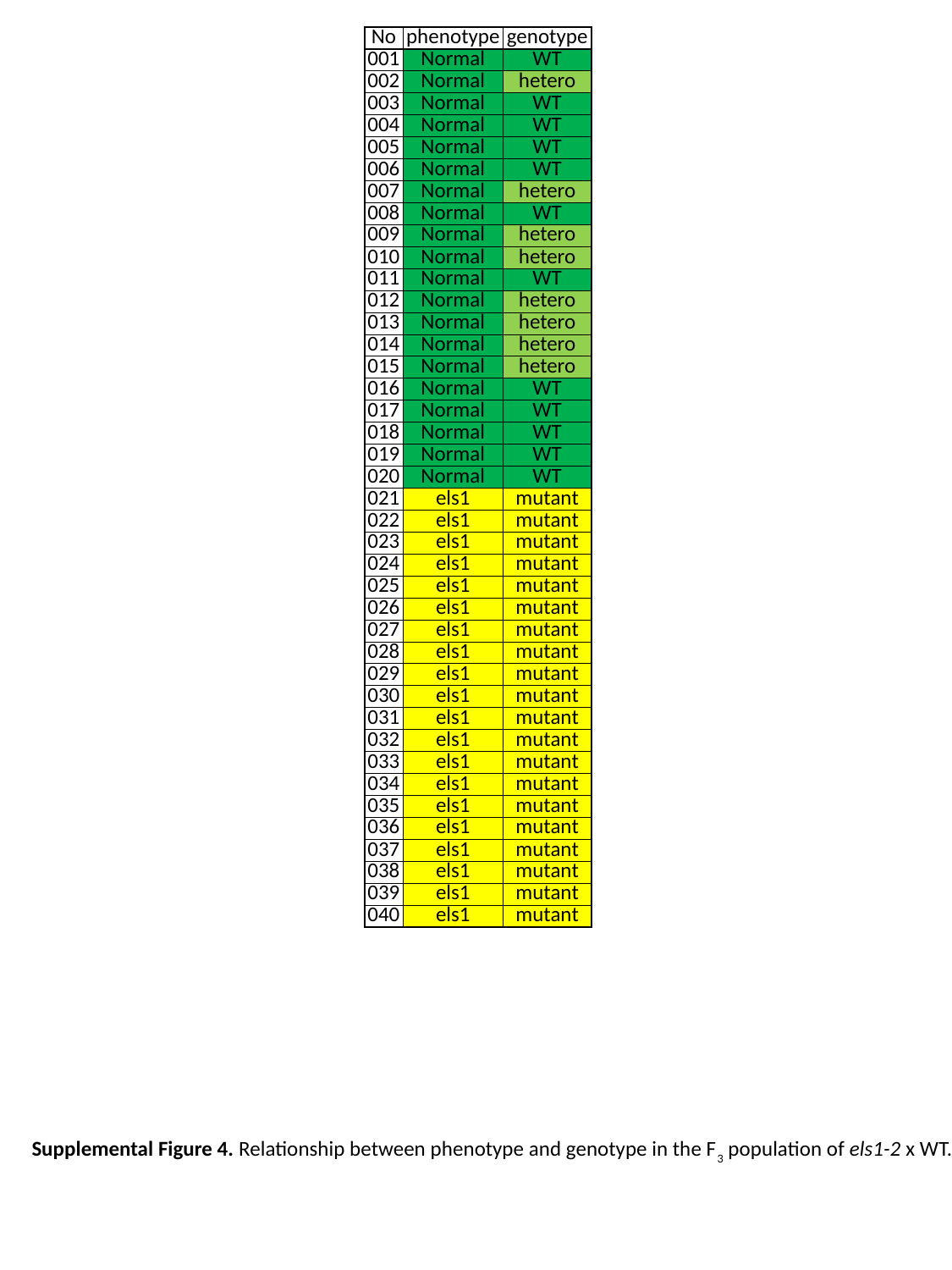

| No | phenotype | genotype |
| --- | --- | --- |
| 001 | Normal | WT |
| 002 | Normal | hetero |
| 003 | Normal | WT |
| 004 | Normal | WT |
| 005 | Normal | WT |
| 006 | Normal | WT |
| 007 | Normal | hetero |
| 008 | Normal | WT |
| 009 | Normal | hetero |
| 010 | Normal | hetero |
| 011 | Normal | WT |
| 012 | Normal | hetero |
| 013 | Normal | hetero |
| 014 | Normal | hetero |
| 015 | Normal | hetero |
| 016 | Normal | WT |
| 017 | Normal | WT |
| 018 | Normal | WT |
| 019 | Normal | WT |
| 020 | Normal | WT |
| 021 | els1 | mutant |
| 022 | els1 | mutant |
| 023 | els1 | mutant |
| 024 | els1 | mutant |
| 025 | els1 | mutant |
| 026 | els1 | mutant |
| 027 | els1 | mutant |
| 028 | els1 | mutant |
| 029 | els1 | mutant |
| 030 | els1 | mutant |
| 031 | els1 | mutant |
| 032 | els1 | mutant |
| 033 | els1 | mutant |
| 034 | els1 | mutant |
| 035 | els1 | mutant |
| 036 | els1 | mutant |
| 037 | els1 | mutant |
| 038 | els1 | mutant |
| 039 | els1 | mutant |
| 040 | els1 | mutant |
Supplemental Figure 4. Relationship between phenotype and genotype in the F3 population of els1-2 x WT.

## Slide 5
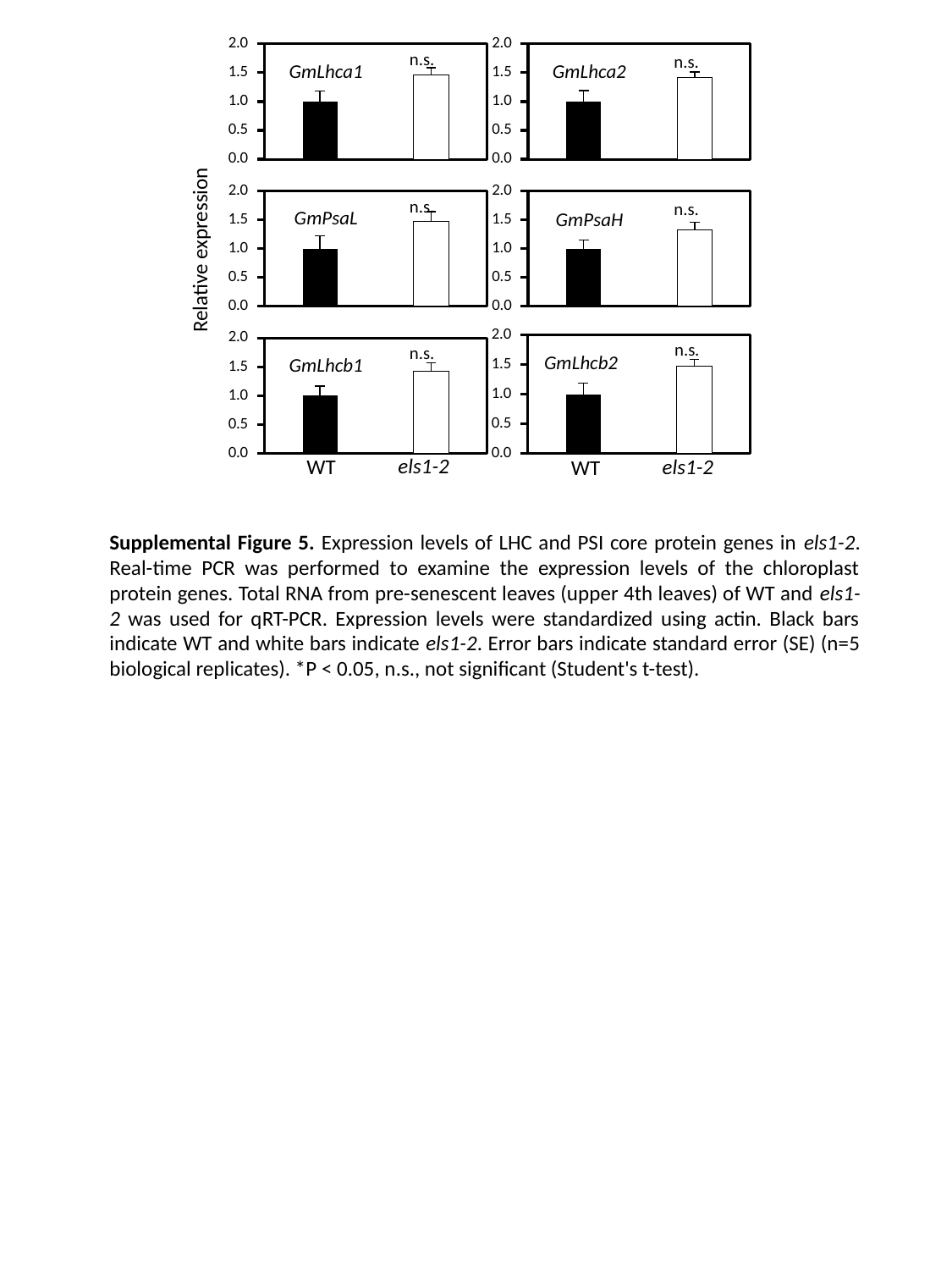

### Chart
| Category | |
|---|---|
| WT | 0.9998130409041103 |
| els1-2 | 1.4635519606813407 |
### Chart
| Category | |
|---|---|
| WT | 1.0001638610289334 |
| els1-2 | 1.4203975555427415 |n.s.
n.s.
GmLhca1
GmLhca2
### Chart
| Category | |
|---|---|
| WT | 0.999825303234271 |
| els1-2 | 1.4777996646824167 |
### Chart
| Category | |
|---|---|
| WT | 1.000194570952106 |
| els1-2 | 1.3190018499312806 |n.s.
n.s.
GmPsaL
GmPsaH
Relative expression
### Chart
| Category | |
|---|---|
| WT | 0.9998701750953283 |
| els1-2 | 1.4703849742374984 |
### Chart
| Category | |
|---|---|
| WT | 1.0001842945689439 |
| els1-2 | 1.429290468888968 |n.s.
n.s.
GmLhcb2
GmLhcb1
els1-2
WT
els1-2
WT
Supplemental Figure 5. Expression levels of LHC and PSI core protein genes in els1-2. Real-time PCR was performed to examine the expression levels of the chloroplast protein genes. Total RNA from pre-senescent leaves (upper 4th leaves) of WT and els1-2 was used for qRT-PCR. Expression levels were standardized using actin. Black bars indicate WT and white bars indicate els1-2. Error bars indicate standard error (SE) (n=5 biological replicates). *P < 0.05, n.s., not significant (Student's t-test).

## Slide 6
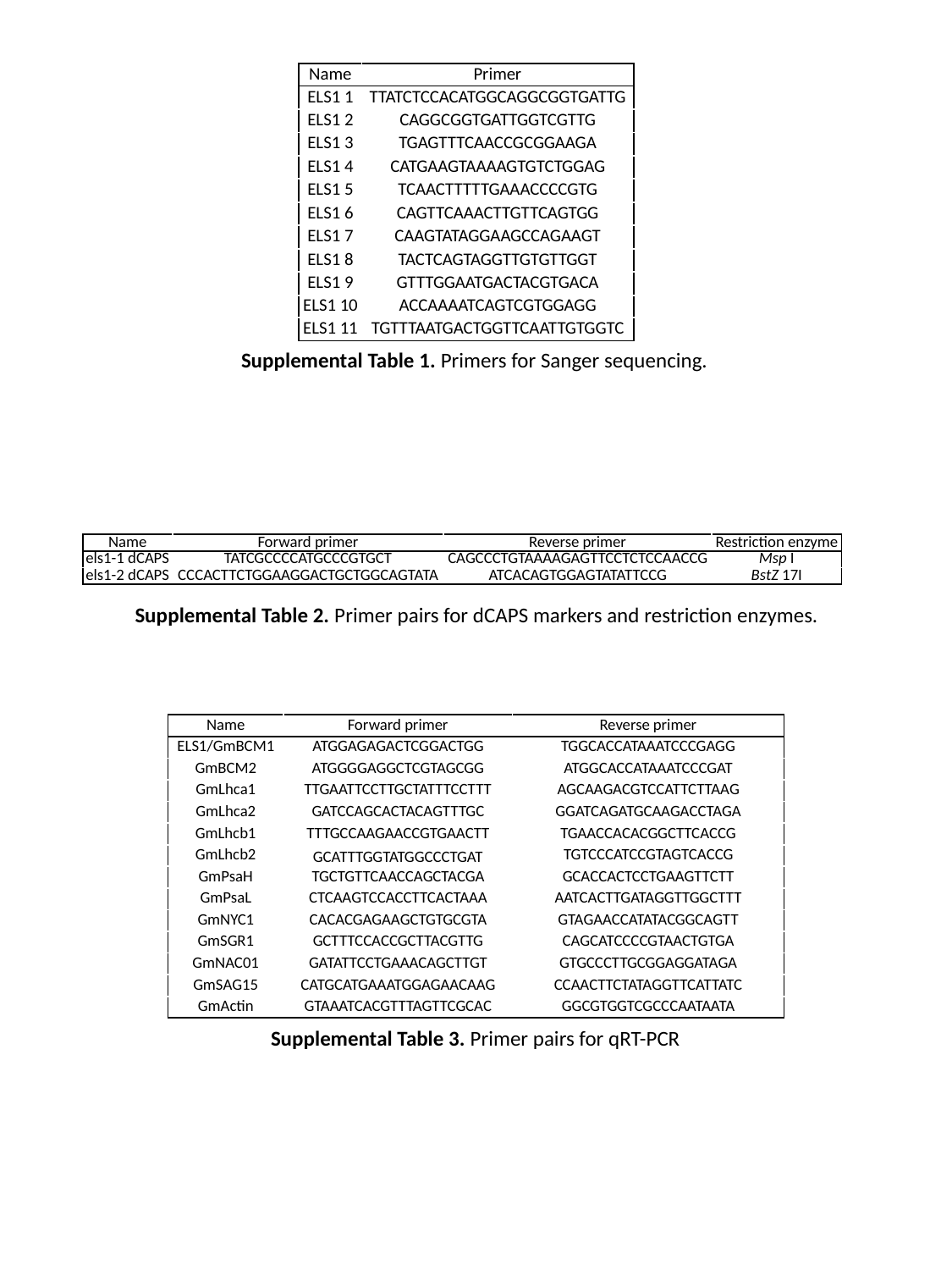

| Name | Primer |
| --- | --- |
| ELS1 1 | TTATCTCCACATGGCAGGCGGTGATTG |
| ELS1 2 | CAGGCGGTGATTGGTCGTTG |
| ELS1 3 | TGAGTTTCAACCGCGGAAGA |
| ELS1 4 | CATGAAGTAAAAGTGTCTGGAG |
| ELS1 5 | TCAACTTTTTGAAACCCCGTG |
| ELS1 6 | CAGTTCAAACTTGTTCAGTGG |
| ELS1 7 | CAAGTATAGGAAGCCAGAAGT |
| ELS1 8 | TACTCAGTAGGTTGTGTTGGT |
| ELS1 9 | GTTTGGAATGACTACGTGACA |
| ELS1 10 | ACCAAAATCAGTCGTGGAGG |
| ELS1 11 | TGTTTAATGACTGGTTCAATTGTGGTC |
Supplemental Table 1. Primers for Sanger sequencing.
| Name | Forward primer | Reverse primer | Restriction enzyme |
| --- | --- | --- | --- |
| els1-1 dCAPS | TATCGCCCCATGCCCGTGCT | CAGCCCTGTAAAAGAGTTCCTCTCCAACCG | Msp I |
| els1-2 dCAPS | CCCACTTCTGGAAGGACTGCTGGCAGTATA | ATCACAGTGGAGTATATTCCG | BstZ 17I |
Supplemental Table 2. Primer pairs for dCAPS markers and restriction enzymes.
| Name | Forward primer | Reverse primer |
| --- | --- | --- |
| ELS1/GmBCM1 | ATGGAGAGACTCGGACTGG | TGGCACCATAAATCCCGAGG |
| GmBCM2 | ATGGGGAGGCTCGTAGCGG | ATGGCACCATAAATCCCGAT |
| GmLhca1 | TTGAATTCCTTGCTATTTCCTTT | AGCAAGACGTCCATTCTTAAG |
| GmLhca2 | GATCCAGCACTACAGTTTGC | GGATCAGATGCAAGACCTAGA |
| GmLhcb1 | TTTGCCAAGAACCGTGAACTT | TGAACCACACGGCTTCACCG |
| GmLhcb2 | GCATTTGGTATGGCCCTGAT | TGTCCCATCCGTAGTCACCG |
| GmPsaH | TGCTGTTCAACCAGCTACGA | GCACCACTCCTGAAGTTCTT |
| GmPsaL | CTCAAGTCCACCTTCACTAAA | AATCACTTGATAGGTTGGCTTT |
| GmNYC1 | CACACGAGAAGCTGTGCGTA | GTAGAACCATATACGGCAGTT |
| GmSGR1 | GCTTTCCACCGCTTACGTTG | CAGCATCCCCGTAACTGTGA |
| GmNAC01 | GATATTCCTGAAACAGCTTGT | GTGCCCTTGCGGAGGATAGA |
| GmSAG15 | CATGCATGAAATGGAGAACAAG | CCAACTTCTATAGGTTCATTATC |
| GmActin | GTAAATCACGTTTAGTTCGCAC | GGCGTGGTCGCCCAATAATA |
Supplemental Table 3. Primer pairs for qRT-PCR

## Slide 7
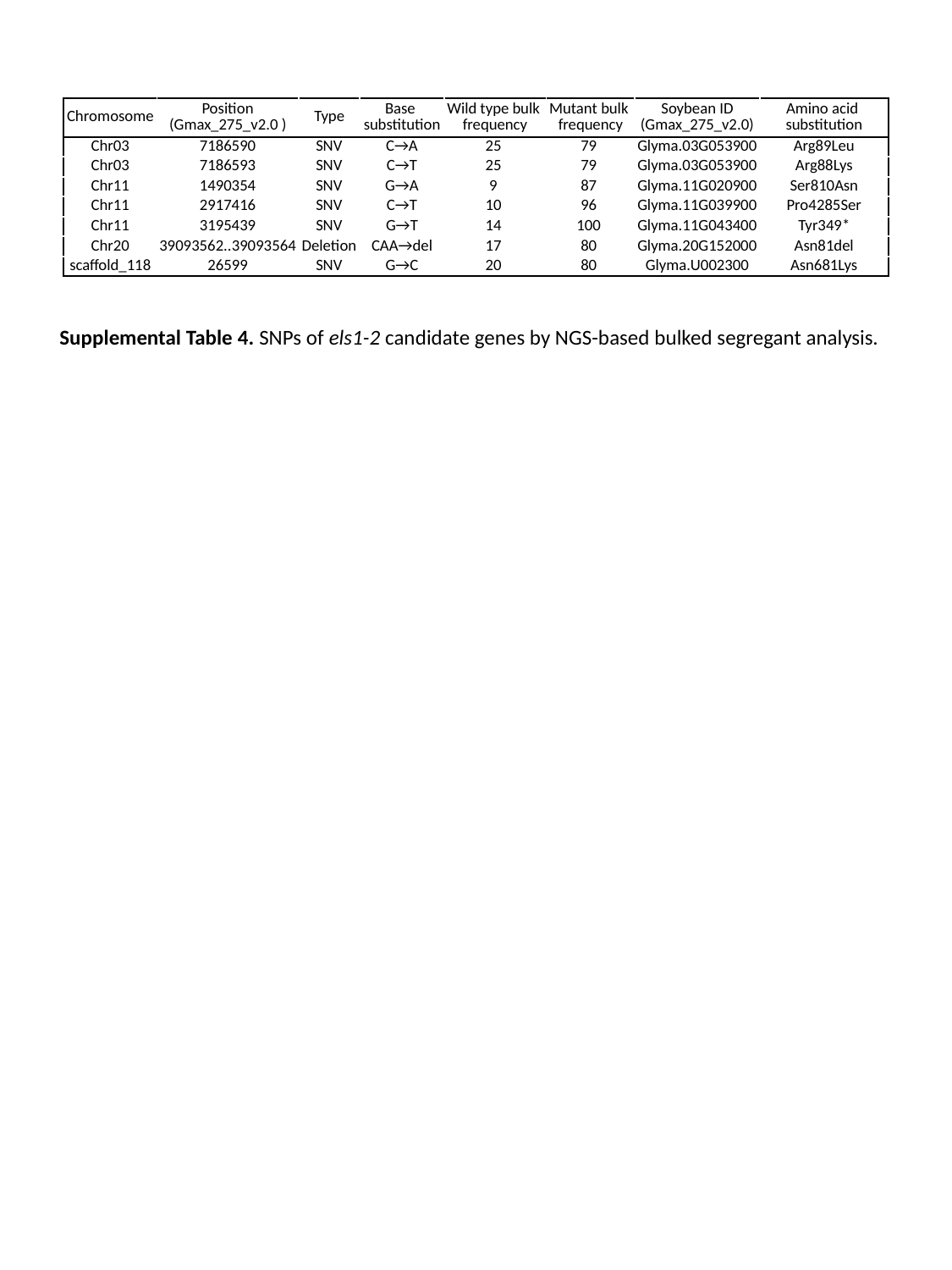

| Chromosome | Position (Gmax\_275\_v2.0 ) | Type | Base substitution | Wild type bulk frequency | Mutant bulk frequency | Soybean ID (Gmax\_275\_v2.0) | Amino acid substitution |
| --- | --- | --- | --- | --- | --- | --- | --- |
| Chr03 | 7186590 | SNV | C→A | 25 | 79 | Glyma.03G053900 | Arg89Leu |
| Chr03 | 7186593 | SNV | C→T | 25 | 79 | Glyma.03G053900 | Arg88Lys |
| Chr11 | 1490354 | SNV | G→A | 9 | 87 | Glyma.11G020900 | Ser810Asn |
| Chr11 | 2917416 | SNV | C→T | 10 | 96 | Glyma.11G039900 | Pro4285Ser |
| Chr11 | 3195439 | SNV | G→T | 14 | 100 | Glyma.11G043400 | Tyr349\* |
| Chr20 | 39093562..39093564 | Deletion | CAA→del | 17 | 80 | Glyma.20G152000 | Asn81del |
| scaffold\_118 | 26599 | SNV | G→C | 20 | 80 | Glyma.U002300 | Asn681Lys |
Supplemental Table 4. SNPs of els1-2 candidate genes by NGS-based bulked segregant analysis.
